# Data Resource Profile: Harmonized health survey data for 240 cities across 11 countries in Latin America: the SALURBAL project

**DOI:** 10.1093/ije/dyae171

**Published:** 2025-01-06

**Authors:** Kari Moore, Mariana Lazo, Ana Ortigoza, D Alex Quistberg, Brisa Sanchez, Binod Acharya, Tania Alfaro, Maria Fernanda Kroker-Lobos, Mariana Carvalho De Menezes, Olga Lucia Sarmiento, Amanda C de Souza Andrade, Carolina Perez Ferrer, Akram Hernandez Vasquez, Waleska Teixeira Caiaffa, Ana V Diez Roux, SALURBAL Group, Marcio Alazraqui, Hugo Spinelli, Carlos Guevel, Vanessa Di Cecco, Adela Tisnés, Carlos Leveau, Adrián Santoro, Damián Herkovits, Andrés Trotta, Patricia Aguirre, Serena Mónica Perner, Santiago Rodríguez López, Natalia Tumas, Nelson Gouveia, Maria Antonietta Mascolli, Anne Dorothée Slovic, Lucas Soriano Martins, Cláudio Makoto Kanai, Maurício Barreto, Gervásio Santos, Anderson Dias de Freitas, Aureliano Sancho Souza Paiva, José Firmino de Sousa Filho, Maria Izabel dos Santos Bell, Roberto Fernandes Silva Andrade, Caio Porto De Castro, Letícia de Oliveira Cardoso, Mariana Carvalho de Menezes, Maria de Fatima Rodrigues Pereira de Pina, Daniel Albert Skaba, Joanna Miguez Nery Guimarães, Vanderlei Pascoal de Matos, Mariana Carvalho de Menezes, Waleska Teixeira Caiaffa, Amélia Augusta de Lima Friche, Carina Maris de Souza, Débora Moraes Coelho, Denise Marques Sales, Guilherme Aparecido Santos Aguilar, Guilherme Ottoni, Julia de Carvalho Nascimento, Lídia Maria de Oliveira Morais, Mariana de Melo Santos, Solimar Carnavalli Rocha, Uriel Moreira Silva, Camila Teixeira Vaz, Amanda Cristina de Souza Andrade, Patricia Frenz, Tania Alfaro, Carolina Nazzal, Cynthia Córdova, Pablo Ruiz, Mauricio Fuentes, Marianela Castillo, Rodrigo Mora, Sebastian Pedrero, Lorena Rodríguez, Sandra Flores, Tamara Doberti, Alejandra Vives Vergara, Alejandro Salazar, Cristián Schmitt, Daniela Olivares, Francisca González, Fernando Baeza, Flavia Angelini, Ignacio Díaz, Laura Orlando, Natalia Díaz, Pablo Campos, Roxana Valdebenito, Victoria León, Andrea Cortinez-O'Ryan, Olga Lucía Sarmiento, Andrés Felipe Aguilar, Julián Arellana, Claudia Bedoya, Jorge Alexander Bonilla, Marcelo Botero, Sergio Cabrales, Germán Carvajal, Natalia Cely, Diego Lucumí Cuesta, Carlos Mauricio Díaz, Karen Fajardo, Catalina González, Silvia Alejandra González, Oscar Guaje, John Alexis Guerra, Paula Guevara, Tomás Guevara Aladino, Luis Ángel Guzmán, Philipp Hessel, Diana Higuera, Bernardo Huertas, Jorge Huertas, Ana Maria Jaramillo, Joaquín Hernando Jaramillo Sabogal, Mario Linares, Julieth Lopez, Diego Lucumí, Paola Martinez, Andrés Medaglia, Daniela Mendez, Ricardo Morales, Felipe Montes, Anamaria Muñoz Florez, Alejandro Palacio, Fabian Camilo Peña, José David Pinzón, Camilo Triana, Andres Felipe Useche, Maria Alejandra Wilches, Sandra Zúñiga, Carlos Moncada, Lina Martínez, Jose David Meisel, Eliana Martinez, María Fernanda Kroker-Lobos, Manuel Ramirez-Zea, Monica Mazariegos, Analí Morales, Tonatiuh Barrientos-Gutierrez, Arantxa Colchero Aragones, Carolina Perez-Ferrer, Francisco Javier Prado-Galbarro, Nancy Paulina López Olmedo, Filipa de Castro, Rosalba Rojas-Martínez, Alejandra Jauregui, Dalia Stern, Horacio Riojas, José Luis Texcalac, Herney Alonso Rengifo Reina, Desirée Vidaña Pérez, Yenisei Ramírez Toscano, J Jaime Miranda, Cecilia Anza-Ramirez, Francisco Diez-Canseco, Akram Hernández Vásquez, Lorena Saavedra-Garcia, Jessica H Zafra-Tanaka, Ross Hammond, Daniel Rodriguez, Maryia Bakhtsiyarava, Iryna Dronova, Xize Wang, Mika Ruchama Moran, Yuanyuan Zhao, Yang Ju, Xavier Delclòs-Alió, Peter Hovmand, Ellis Ballard, Jill Kuhlberg, Ana Diez Roux, Binod Acharya, Amy Auchincloss, Ione Avila-Palencia, Sharrelle Barber, Usama Bilal, Ariela Braverman, Dustin Fry, Felipe Garcia-España, Katherine Indvik, Josiah Kephart, Carolyn Knoll, Brent Langellier, Mariana Lazo, Ran Li, Gina Lovasi, Rosie Mae Henson, Kevin Martinez-Folgar, Steve Melly, Yvonne Michael, Kari Moore, Jeff Moore, Pricila Mullachery, Ana Ortigoza, Harrison Quick, D Alexander Quistberg, Jordan Rodriguez Hernandez, Brisa Sanchez, S Claire Slesinski, Ivana Stankov, Jose Tapia Granados, Bricia Trejo, Goro Yamada

**Affiliations:** Urban Health Collaborative, Dornsife School of Public Health, Drexel University, Philadelphia, PA, USA; Urban Health Collaborative, Dornsife School of Public Health, Drexel University, Philadelphia, PA, USA; Department of Community Health and Prevention, Dornsife School of Public Health, Drexel University, Philadelphia, PA, USA; Urban Health Collaborative, Dornsife School of Public Health, Drexel University, Philadelphia, PA, USA; Department of Social and Environmental Determinants for Health Equity, Pan American Health Organization, Washington, DC, USA; Urban Health Collaborative, Dornsife School of Public Health, Drexel University, Philadelphia, PA, USA; Department of Environmental and Occupational Health, Dornsife School of Public Health, Drexel University, Philadelphia, PA, USA; Urban Health Collaborative, Dornsife School of Public Health, Drexel University, Philadelphia, PA, USA; Department of Epidemiology and Biostatistics, Dornsife School of Public Health, Drexel University, Philadelphia, PA, USA; Urban Health Collaborative, Dornsife School of Public Health, Drexel University, Philadelphia, PA, USA; Programa de Doctorado en Salud Pública, Escuela de Salud Pública, Facultad de Medicina, Universidad de Chile, and Center for Cancer Prevention and Control (CECAN), Santiago, Chile; Centro de Investigación del INCAP para la Prevención de Enfermedades Crónicas (CIIPEC), Instituto de Nutrición de Centro América y Panamá, Ciudad de Guatemala, Guatemala; Department of Clinical and Social Nutrition, School of Nutrition, Federal University of Ouro Preto, Ouro Preto, Minas Gerais, Brazil; School of Medicine, Universidad de Los Andes, Bogotá, Colombia; Instituto de Saúde Coletiva, Universidade Federal de Mato Grosso, Cuiabá, Mato Grosso, Brazil; Center for Nutrition and Health Research, Instituto Nacional de Salud Pública, Cuernavaca, Morelos, Mexico; National Council for Science and Technology, Mexico City, Mexico; CRONICAS Centre of Excellence in Chronic Diseases, Universidad Peruana Cayetano Heredia, Lima, Peru; Observatory for Urban Health in Belo Horizonte, Federal University of Minas Gerais, Belo Horizonte, Brazil; Urban Health Collaborative, Dornsife School of Public Health, Drexel University, Philadelphia, PA, USA; Department of Epidemiology and Biostatistics, Dornsife School of Public Health, Drexel University, Philadelphia, PA, USA

**Keywords:** Health survey, Latin America, socioeconomic, health risk factors, urban health

Key Features

- Harmonized health data sources in Latin America for research and action are limited. The SALURBAL (Salud Urbana en America Latina/Urban Health in Latin America) health survey data resource was created as part of an integrated, comprehensive resource to characterize and study the drivers of urban health in middle-income countries in Latin America. This resource is the largest in scope and size in cities in Latin America. It includes harmonized health survey data from 40 surveys from 11 countries. Survey years ranged from 2000 to 2021, the range of surveys per country is one to six, with seven out of the 11 countries having more than one survey. The total number of adults included in the resource is 721 099 (with sociodemographic data) and 542 336 with select health-related outcomes. For children (aged 0–17), the samples are 209 379 and 134 833, respectively.
- Data available for adults include individual-level demographic and socioeconomic characteristics, alcohol/tobacco use, anthropometry, diet, physical activity, diabetes, hypertension, mental health and self-reported health for adults. For children, data include household-level demographic and socioeconomic characteristics and individual-level anthropometry.
- City-level, model-based prevalence estimates for selected health risk factors are available as part of this resource.
- This data resource can be linked to built, natural and social environment data for select geographies [cities, subcity units (e.g. counties) and neighbourhoods).
- Researchers interested in the SALURBAL project data should visit: [https://data.lacurbanhealth.org].

## Data resource basics

Focused on one of the most highly urbanized regions of the world, Latin America, the SALURBAL (Salud Urban en America Latina/Urban Health in Latin America) Project was launched in 2017 to build a novel and unique multi-national, multi-level, multi-dimensional data infrastructure to allow the investigation of questions about how urban environments and policies affect health, health equity and environmental sustainability and to serve as a platform for research and action in the region.^1^^,^^2^

A detailed description of the SALURBAL project is available elsewhere.^1^^,^^2^ Briefly, the SALURBAL includes data from all urban agglomerations (‘cities’, total 371) with 100 000 residents or more in 2010 in 11 Latin American countries (Argentina, Brazil, Chile, Colombia, Costa Rica, El Salvador, Guatemala, Mexico, Nicaragua, Panama, and Peru). Of the 371 cities, 240 had health survey data available (Supplementary Figure S1, available as Supplementary data at *IJE* online). SALURBAL pooled and harmonized surveys and linked individual-level data to social, built and natural environment data for specific geographies, including countries, cities, sub-cities and ‘neighbourhoods’. SALURBAL defined cities as agglomerations of administrative units (i.e. municipios, comunas etc.) that encompass a city’s built-up area as defined using visual inspection of satellite images.^2^ These administrative units are referred to as sub-city units. ‘Neighbourhoods’ were defined as the smallest unit for which census data were available, similar to US census tracts (i.e. radio censal, sector censitario, zona censal, area geoestadistica basica, barrio etc.).

The SALURBAL health survey component includes harmonized data from 40 national surveys from 11 countries, including 13 surveys (from seven countries) with data for children, with survey years ranging from 2000 to 2021. Another component included in this resource is model-based city-level prevalence estimates of selected health risk factors.

## Data collected

### Numbers of surveys, cities and participants

The SALURBAL health survey component compiled, linked and harmonized data from 40 national surveys from 11 countries (Table 1; Supplementary Table S1 and Figure S2, available as Supplementary data at *IJE* online), including 13 surveys (from seven countries) with data for children. The majority of the data corresponds to surveys conducted between 2010 and 2021. The range of surveys per country is one to six, allowing for trend analyses (i.e. changes over time using repeat cross-sectional data) (see Data Resource Use section below). The total number of adults included in the resource is 721 099 (with sociodemographic data) and 542 336 with selected health-related outcomes. For children (aged 0–17), the samples are 209 379 and 134 833, respectively. A total of 240 unique SALURBAL-defined cities having at least one survey respondent is represented in the resource. The criteria for inclusion of surveys in the harmonized survey resource are shown in Supplementary Figure S3 (available as Supplementary data at *IJE* online) and described below.

**Table 1:** Summary of health surveys included in Salud Urbana en America Latina/Urban Health in Latin America (SALURBAL) data resource, by country name (alphabetical order) and survey year. For detailed characteristics, please see Supplementary Table S1 (available as Supplementary data at *IJE* online)

Country	Survey name	Survey year	Child data	*N* in SALURBAL cities	Age (years)
Argentina	Encuesta Nacional de Factores de Riesgo, ENFR (National Risk Factors Survey)	2005	No	25 753	≥18
Argentina	Encuesta Nacional de Factores de Riesgo, ENFR (National Risk Factors Survey)	2009	No	16 218	≥18
Argentina	Encuesta Nacional de Factores de Riesgo, ENFR (National Risk Factors Survey)	2013	No	21 451	≥18
Brasil	Pesquisa Nacional de Saúde, PNS (National Health Survey)	2013	No	29 353 in L2s;	≥18
				40 703 in L1ADs	
Brasil	Pesquisa Nacional de Saúde, PNS (National Health Survey)	2019	No	33 515 in L2s;	≥18
				46 767 in L1ADs	
Chile	Encuesta Nacional de Salud, ENS (National Health Survey)	2003	No	2032	≥17
Chile	Encuesta Nacional de Salud, ENS (National Health Survey)	2010	No	3140	≥15
Chile	Encuesta Nacional de Salud, ENS (National Health Survey)	2016–17	No	3805	≥15
Chile	Encuesta Longitudinal de Primera Infancia (ELPI) (Longitudinal Survey of Early Childhood)	2017–18	Yes	6723	1–12
Colombia	Encuesta Nacional de Salud, ENS (National Health Survey)	2007	No	43 182	18–64
Colombia	Encuesta Nacional de la Situation Nutricional en Colombia, ENSIN (National Nutritional Situation in Colombia)	2005	Yes	42 336 adults	0–64
				(18–69);	
				23 794 children	
				(<18)	
Colombia	Encuesta Nacional de la Situation Nutricional en Colombia, ENSIN (National Nutritional Situation in Colombia)	2010	Yes	55 863 adults	0–64
				(18–69);	
				30 278 children	
				(<18)	
Colombia	Encuesta Nacional de la Situation Nutricional en Colombia, ENSIN (National Nutritional Situation in Colombia)	2015	Yes	36 593 adults	0–64
				(18–69);	
				17 104 children	
				(<18)	
Costa Rica	Encuesta Multinacional de Diabetes mellitus y Factores de Riesgo, CAMDI (Multinational Survey of Diabetes Mellitus & Risk Factors, Central American Diabetes Initiative)	2005	No	1427	≥20
Guatemala	Encuesta Multinacional de Diabetes mellitus y Factores de Riesgo, CAMDI (Multinational Survey of Diabetes Mellitus & Risk Factors, Central American Diabetes Initiative)	2002–03	No	1397	≥20
Guatemala	Demographic and Health Survey (DHS)	2014–15	Yes	2730 adult females (18–49); 983 children (<5)	Females 18–49; children <5
Nicaragua	Encuesta Multinacional de Diabetes mellitus y Factores de Riesgo, CAMDI (Multinational Survey of Diabetes Mellitus & Risk Factors, Central American Diabetes Initiative)	2003	No	1397	≥20
Mexico	Encuesta National de Salud, ENSA (National Health Survey)	2000	Yes	29 733 adults (≥18); 23 966 children (<5)	Adults ≥18; children <5
Mexico	Encuesta Nacional de Salud y Nutricion, ENSANUT (National Survey for Health and Nutrition)	2006	Yes	31 532 adults (≥18); 19 431 children (<18)	All ages
Mexico	Encuesta Nacional de Salud y Nutricion, ENSANUT (National Survey for Health and Nutrition)	2012	Yes	26 335 adults (≥18); 25 014 children (<18)	All ages
Mexico	Encuesta Nacional de Salud y Nutricion, ENSANUT (National Survey for Health and Nutrition)	2016	Yes	14 618 adults (≥18); 3274 children (<18)	All ages
Mexico	Encuesta Nacional de Salud y Nutricion, ENSANUT (National Survey for Health and Nutrition)	2018	Yes	27 118 adults (≥18); 7538 children (<18)	All ages
Panama	Encuesta Nacional de Salud y Calidad de Vida ENSCAVI (National Survey of Health and Quality of Life)	2007	No	11 394	≥18 years
Peru	Encuesta Nacional de Demografia y Salud, ENDES (National Survey of Demographics and Health)	2016	Yes	11 929 adults (≥18); 8547 children (<5)	Adults ≥15 years; children <5 years
El Salvador	Encuesta Multinacional de Diabetes mellitus y Factores de Riesgo, CAMDI (Multinational Survey of Diabetes Mellitus & Risk Factors, Central American Diabetes Initiative)	2004	No	1872	≥20
El Salvador	Encuesta Nacional de Salud Familiar, FESAL (National Family Health Survey)	2008	Yes	4297 adult females (18–49); 1290 children (<5)	Females 18–49 years; children <5 years
El Salvador	Encuesta Nacional de Enfermedades Cronicas no transmisibles en Poblacion Adulta de El Salvador ENECA (National Survey of Noncommunicable Chronic Diseases in the Adult Population of El Salvador)	2014–15	No	1 546	≥20

CAMDI, Encuesta Multinacional de Diabetes mellitus y Factores de Riesgo (Multinational Survey of Diabetes Mellitus & Risk Factors, Central American Diabetes Initiative); DHS, Demographic and Health Survey, ELPI, Encuesta Longitudinal de Primera Infancia (Longitudinal Survey of Early Childhood); ENDES, Encuesta Nacional de Demografia y Salud (National Survey of Demographics and Health); ENECA, Encuesta Nacional de Enfermedades Cronicas no transmisibles en Poblacion Adulta de El Salvador (National Survey of Noncommunicable Chronic Diseases in the Adult Population of El Salvador); ENFR, Encuesta Nacional de Factores de Riesgo (National Risk Factors Survey); ENS, Encuesta Nacional de Salud (National Health Survey); ENSA, Encuesta National de Salud (National Health Survey); ENSANUT, Encuesta Nacional de Salud y Nutricion (National Survey for Health and Nutrition); ENSCAVI, Encuesta Nacional de Salud y Calidad de Vida (National Survey of Health and Quality of Life); ENSIN, Encuesta Nacional de la Situation Nutricional en Colombia (National Nutritional Situation in Colombia); FESAL, Encuesta Nacional de Salud Familiar (National Family Health Survey); L1AD, Level 1 administrative cities; L2, Level 2 administrative subcities; PNS, Pesquisa Nacional de Saúde (National Health Survey); SALURBAL, (Salud Urbana en America Latina/Urban Health in Latin America).

### Data resource production

In this section we first describe the harmonization process and then the production of model-based prevalence estimates.

The SALURBAL Health Survey data harmonization efforts were led by the SALURBAL Data and Methods Core with leadership at Drexel University and members from country teams. For the creation of this resource, SALURBAL followed four guiding principles: **(**i) use existing national health survey data administered by agencies within each country; (ii) restrict to surveys with geographical information that could be linked to SALURBAL sub-city level, publicly or through request to the agency implementing the survey; (iii) prioritize surveys with information on non-communicable health behaviours and risk factors; (iv) use of harmonization approaches that are rigorous but flexible to accommodate differences across surveys, described in more detail below.^3^^,^^4^

#### Survey identification and confirmation of initial screening of eligibility criteria

SALURBAL country teams identified national health surveys within each country and confirmed whether they met the inclusion criteria related to geographical linkage potential and focus (Supplementary Figure S3, available as Supplementary data at *IJE* online).

#### Survey data acquisition

Survey data were gathered from national bureaus of statistics or other relevant government ministries or agencies responsible for the survey use. Data were gathered either by directly downloading from the agencies’ public websites when available or by making formal requests to the agency.

#### Linkage to SALURBAL geographies

Once the data were gathered, we reviewed the data to ensure that geographical identifiers were available to link to the SALURBAL-defined sub-city or neighbourhood level. Only surveys that contained the necessary geographical linkages were further reviewed.

#### Survey content review

Next, the survey forms and metadata were reviewed to determine if they contained information for at least one pre-selected non-communicable disease risk factors (anthropometry, diabetes, hypertension, tobacco use, alcohol use, diet, physical activity) and key sociodemographic information (age, sex or gender and educational attainment).

#### Harmonization approach

First, we identified and collated the survey questions and response options by domain (Supplementary Figure S4 and Supplementary Table S2, available as Supplementary data at *IJE* online). We determined all skip patterns in the questions and respondent universe for each relevant question. For some questions, only a subset of respondents was included, such as a specified age range or sex/gender, or a randomly selected subset of the survey respondents. Because the surveys were administered in Spanish or Portuguese, the next step was translating into English as a common language. This was accomplished via project members who were fluent in both languages. Once the questions available were compiled by domain, we reviewed recommended definitions used by others such as the Centers for Disease Control and Prevention (CDC) or the World Health Organization (WHO).

As part of harmonization, we addressed the following three major challenges:

- discrepancy in sets of questions, scales and questionnaires used for retrieving information about similar health behaviours or health outcomes, e.g. diabetes, hypertension, physical activity, diet, alcohol intake;
- lack of consistency in response categories or measurement units used. eg self-rated health;
- differences in respondent universe for selected health outcomes, e.g. gestational diabetes, use of medications for hypertension/diabetes, doctor/physician diagnosis of depression.

To address these challenges, we used a flexible approach focused on creating different versions of variables based on scenarios. Surveys with questions that were asked in a similar manner in terms of response patterns and wording of the questions were grouped together as a scenario. For example, when harmonizing diabetes, some surveys asked, ‘Have you ever been diagnosed with diabetes or high blood sugar by a doctor?’ with response options of ‘yes’ and ‘no’, followed by an additional question (‘was this diagnosis during pregnancy?’) to women who responded ‘yes’ to the initial diabetes question. Other surveys asked the same leading question but included three response options of ‘yes’, ‘no’ and ‘yes, but only during pregnancy’, with no further questions. These were categorized into two different scenarios. Once the questions were grouped into scenarios, we developed three different approaches to harmonization, which we refer to as harmonization types. Type 1 occurred when surveys had questions that were asked in the same way with only slight differences in wording, such that there was only one scenario and it was possible to create a single harmonized version of the measure. Examples of this harmonization type are sex and depressive symptoms scales. Type 2 occurred when the survey questions were asked in different ways in terms of wording, response options and response patterns availabl,e such that there were multiple question scenarios but it was still possible to identify one harmonized version of the measure. Examples of this harmonization type are marital status and educational attainment. Type 3 occurred when multiple versions of the measure were created because we could not harmonize to a common definition, due to differences in the question wording. Examples of this harmonization type are hypertension and binge drinking.

All proposed harmonization was reviewed by country teams and working groups with expertise in the domain, and all harmonization procedures are detailed in written protocols that accompany the datasets (see section below).

After applying the harmonization protocol to the datasets, we reviewed descriptive statistics of initial harmonized variables. If any deviations from expected results (i.e. known patterns) were observed, we reviewed all programming and corrected errors. If deviations remained, we reviewed the harmonization protocol and modifications were made as needed.

### Model-based prevalence estimates

Whereas most of the surveys included in SALURBAL were based on complex, multi-stage probability designs to select nationally representative samples of the population of the country and provide nationally representative estimates, these data cannot be used directly to draw inferences about prevalence of diseases or exposures in other geographical units, for example SALURBAL cities. To address that issue and be able to provide city-specific prevalence estimates, SALURBAL employed a model-based prevalence estimates approach. Briefly, sex- and city-specific smoothed estimates were derived by combining information from the sample in the city itself with information from other cities in the same country, using a mixed model with city-specific random effects. Smoothed estimates provide more stable estimates for the smaller cities. The approach also allows standardizing the estimates to other populations (e.g. the SALURBAL population’s age and sex distribution). This approach is similar to Quick *et al*.^5^ 2020, and details are found in the supplementary file.

**Figure 1:**
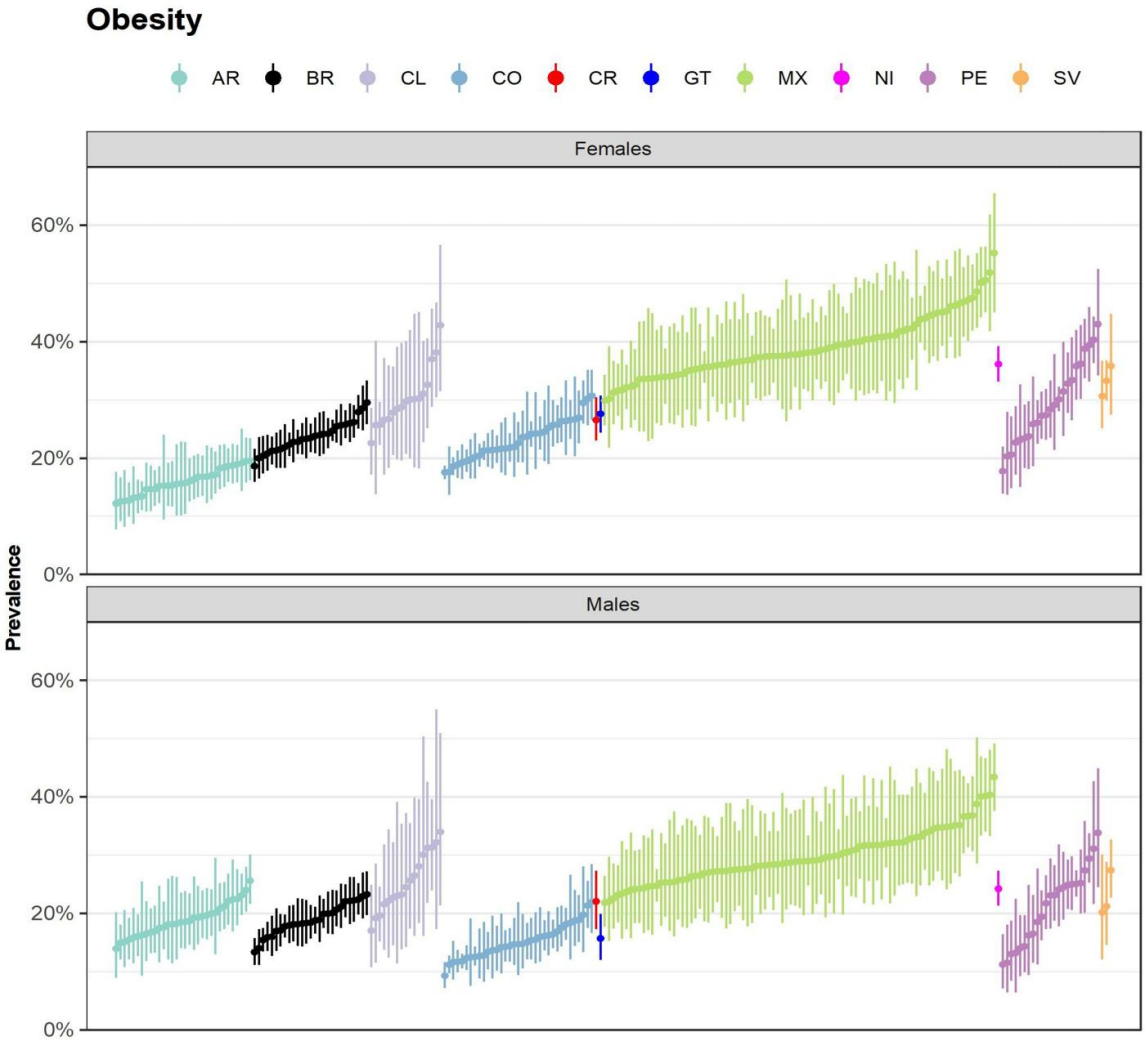
Estimated prevalence of adult (20 years and older) obesity for SALURBAL cities and countries, by sex. AR, Argentina; BR, Brazil; CL, Chile; CO, Colombia; CR, Costa Rica; GT, Guatemala; MX, Mexico; NI, Nicaragua; PE, Peru; SV, El Salvador

Figure 1 displays the prevalence estimates of adult obesity for SALURBAL cities and countries. Additional measures for prevalence of overweight, diabetes, hypertension, poor/fair self-rated health status and current smoking are shown in Supplementary Figures S5–S9 (available as Supplementary data at *IJE* online). In Supplementary Figure S10 (available as Supplementary data at *IJE* online) we used Mexico’s five waves of surveys as an example of the availability and potential use of prevalence estimates over time. For this example, we selected cities that were sampled in each of the waves of Encuesta Nacional de Salud y Nutricion (ENSANUT; a total of 46 cities) and provide sex-stratified, city- and country-level obesity prevalence estimates.

### Variables included in the datasets and documentation production

To accompany all the SALURBAL survey-related datasets, 21 domain-specific harmonization protocols were prepared.. These protocols provide information about the domain, harmonized variable definitions, recommendations, summary of the survey questions used in harmonization, availability across surveys, variables constructions, limitations and references. Appendices of the documents include country-specific questions in both English and the original language, response categories and coding instructions by country. Table 2 summarizes the survey domains and overall availability across surveys. Supplementary Tables S2 and S3 (available as Supplementary data at *IJE* online) show the list of variables harmonized to date. Documentation related to model-based prevalence estimates includes information about the different model-based estimates, their recommended use and interpretation and the methods.

**Table 2:** Summary of harmonized survey domains and main analytical variables available in Salud Urbana en America Latina/Urban Health in Latin America (SALURBAL). For detailed characteristics of main analytical variables, please see Supplementary Table S2 (available as Supplementary data at *IJE* online)

Domain	Number of main analytical variables^a^	% of surveys with all the analytical variables ‘complete harmonization’	% of surveys with at least one harmonized variable in the domain
Adults			
Demographics	6	64%	100%
Socioeconomic status	13	67%	92%
Education	2	100%	100%
Alcohol intake	6	33%	81%
Tobacco use	8	26%	92%
Anthropometry	6	54%	96%
Diet	9	50%	69%
Physical activity	14	34%	62%
Diabetes	3	73%	81%
Hypertension	7	64%	81%
Depressive symptoms	23	12%	50%
Self-reported health status	3	22%	62%
Health care	4	41%	46%
Pregnancy	2	46%	62%
Violence	3	13%	19%
Children			
Demographics	5	68%	100%
Socioeconomic status	16	85%	100%
Anthropometry	8	99%	100%

a Only main analytical variables are included in the creation of this table, see Supplementary Table S2 (available as Supplementary data at *IJE* online) for the list.

### Linkages to other data

Due to the retrospective nature of data collected from secondary sources, not all SALURBAL health survey data can be linked to all SALURBAL geographical levels (city, sub-city and neighbourhood). A summary of the linkage of survey respondents to the different SALURBAL geographical levels is shown in Table 3 and demonstrates heterogeneity in the geolinkage. For example, in the case of Brazil, geolinkage to subcities (municipalidades) was only available for the capital of each of the 27 states, leading to the availability of only 27 cities for the entire country. The linkage of survey participants to ‘neighbourhoods’, the smallest SALURBAL geographical unit, was possible in 11 (39%) of the surveys (four countries).

**Table 3:** Linkage of Salud Urbana en America Latina/Urban Health in Latin America (SALURBAL) Health Survey respondents to SALURBAL ‘Cities’ (SALURBAL Level 1), ‘Subcities’ (SALURBAL Level 2), and ‘Neighbourhoods’ (SALURBAL Level 3)

	SALURBAL ‘Cities’			SALURBAL ‘Subcities’			SALURBAL ‘Neighborhoods’		
Survey	# Cities in SALURBAL	# Cities with survey respondents (%)	Distribution of survey respondents^a^ per City, P50 (P25, P75^a^)	# Subcities units in SALURBAL	# Subcities with survey respondents (%)	Distribution of survey respondents^a^ per Subcity unit, P50 (P25, P75^a^)	# Neighbourhoods in SALURBAL	# Neighbourhoods with survey respondents (%)	Distribution of survey respondents^a^ per Neighbourhood, P50 (P25, P75^a^)
Argentina ENFR 2005	33	32 (97.0%)	783 (379–1214)	110	89 (80.9%)	116 (38–459)	29 792	2051 (6.9%)	11 (6–18)
Argentina ENFR 2009	33	33 (100%)	562 (233–721)	110	109 (99.1%)	109 (41–352)	29 792	1679 (5.6%)	9 (5–14)
Argentina ENFR 2013	33	33 (100%)	511 (417–693)	110	108 (98.2%)	85.5 (50–339.5)	29 792	2698 (9.1%)	8 (6–10)
Brazil PNS 2013	152	27 (17.8%)	927 (834–1179)	422	27 (6.4%)	927 (834–1179)	164 107	Unable to link to L3	
Chile ENS 2003	21	21 (100%)	64 (36–78)	81	67 (82.7%)	18 (11–53)	3913	Unable to link to L3	
Chile ENS 2010	21	21 (100%)	90 (34–184)	81	72 (86.4%)	22.5 (15–53)	3913	528 (13.5%)	6 (4–7)
Chile ENS 2017	21	21 (100%)	133 (72–237)	81	75 (92.6%)	28 (14–70)	3913	753 (19.2%)	4 (2–6)
Chile ELPI 2017	21	6 (21.2%)	214 (187–266)	81	41 (51.2%)	107 (81–187)	3913	Unable to link to L3	
Colombia ENS 2007	35	33 (94.3%)	1044 (468–1737)	84	57 (67.9%)	468 (186–997)	3781	1151 (30.4%)	26 (17–38)
Colombia ENSIN 2005	35	33 (94.3%)	Ages 0–17: 608 (246–920); ages 18–69: 957 (485–1439)	84	57 (67.9%)	Ages 0–17: 241 (105: 608); ages 18–69: 390 (178–1089)	3781	400 (10.6%)	Ages 0–17: 12 (7–20); ages 18–69: 25 (16–32)
Colombia ENSIN 2010	35	35 (100%)	Ages 0–17: 608 (279–1039); ages 18–64: 936 (614–2006)	84	67 (79.8%)	Ages 0–17: 259 (129–558); ages 18–64: 377 (218–942)	3781	1292 (34.2%)	Ages 0–17: 15 (9–23); ages 18–64: 27 (21–39)
Colombia ENSIN 2015	35	35 (100%)	Ages 0–17: 400 (119–583); ages 18–64: 739 (226–1346)	84	68 (81.0%)	Ages 0–17: 62 (36–219); ages 18–64: 221 (137–741)	3781	1375 (36.4%)	Ages 0–17: 6 (3–9); ages 18–64: 22 (14–33)
Costa Rica CAMDI 2005	1	1 (100%)	1427	29	Unable to link to L2		NA	Unable to link to L3	
Guatemala CAMDI 2002	3	1 (33.3%)	1397	20	1 (5.0%)	1397	4025	Unable to link to L3	
Guatemala DHS 2015	3	1 (33.3%)	Ages 0–4: 983; Females 18–49: 2730	20	1 (5.0%)	1161	4025	Unable to link to L3	
Nicaragua CAMDI 2003	5	1 (20.0%)	1993	11	1 (9.1%)	1993	NA		
Mexico ENSA 2000	92	77 (83.7%)	Ages 0–17: 105 (58–223); ges 18+: 207 (107–496)	406	153 (37.7%)	Ages 0–17: 51 (41–101); ages 18+: 153 (37.7%)	32 927	Unable to link to L3	
Mexico ENSANUT 2006	92	89 (96.7%)	Ages 0–17: 134 (67–306); Ages 18+: 179 (72–427)	406	Unable to link to L2		32 927	Unable to link to L3	
Mexico ENSANUT 2012	92	91 (98.9%)	Ages 0–17: 139 (76–302); Ages 18+: 190 (89–388)	406	245 (60.3%)	Ages 0–17: 52 (31–101); ages 18+: 68 (35–137)	32 927	921 (2.8%)	Ages 0–17: 21 (15–29); ages 18+: 32 (28–34)
Mexico ENSANUT 2016	92	59 (64.1%)	Ages 0–17: 38 (21–60); Ages 18+: 41 (27–73)	406	112 (27.6%)	Ages 0–17: 26 (15–37); ages 18+: 35 (25–56)	32 927	189 (0.6%)	Ages 0–17: 15 (8–24); ages 18+: 26 (18–33)
Mexico ENSANUT 2018	92	91 (98.9%)	Ages 0–17: 170 (83–412); ages 18+: 188 (73–368)	406	290 (71.4%)	Ages 0–17: 19 (10–46); ages 18+: 44 (19–100)	32 927	Unable to link to L3	
Panama ENSCAVI 2007	3	3 (100%)	1773 (1738–7883)	82	64 (78.0%)	124 (59–209)	1792	Unable to link to L3	
Peru ENDES 2016	23	23 (100%)	Ages 0–17: 338 (163–400); ages 18+: 373 (168–660)	169	150 (88.8%)	Ages 0–17: 42 (18–79); ages 18+: 51 (20–114)	NA		
El Salvador CAMDI 2004	3	1 (33.3%)	1872	22	1 (4.5%)	1872	NA		
El Salvador FESAL 2008	3	3 (100%)	Ages 0–4: 162 (133–995); females 18–49: 805 (735–5810)	22	21 (95.5%)	Ages 0–4: 43 (28–102); females 18–49: 9 (71–280)	NA		
El Salvador ENECA 2014	3	3 (100%)	352 (164–1030)	22	16 (72.7%)	68 (38–139)	NA		

CAMDI, Encuesta Multinacional de Diabetes mellitus y Factores de Riesgo (Multinational Survey of Diabetes Mellitus & Risk Factors, Central American Diabetes Initiative); DHS, Demographic and Health Survey, ELPI, Encuesta Longitudinal de Primera Infancia (Longitudinal Survey of Early Childhood); ENDES, Encuesta Nacional de Demografia y Salud (National Survey of Demographics and Health); ENECA, Encuesta Nacional de Enfermedades Cronicas no transmisibles en Poblacion Adulta de El Salvador (National Survey of Noncommunicable Chronic Diseases in the Adult Population of El Salvador); ENFR, Encuesta Nacional de Factores de Riesgo (National Risk Factors Survey); ENS, Encuesta Nacional de Salud (National Health Survey); ENSA, Encuesta National de Salud (National Health Survey); ENSANUT, Encuesta Nacional de Salud y Nutricion (National Survey for Health and Nutrition); ENSCAVI, Encuesta Nacional de Salud y Calidad de Vida (National Survey of Health and Quality of Life); ENSIN, Encuesta Nacional de la Situation Nutricional en Colombia (National Nutritional Situation in Colombia); FESAL, Encuesta Nacional de Salud Familiar (National Family Health Survey); L1AD, Level 1 administrative cities; L2, Level 2 administrative subcities; L3, Level 3 administrative neighborhoods; PNS, Pesquisa Nacional de Saúde (National Health Survey); SALURBAL, (Salud Urbana en America Latina/Urban Health in Latin America).

A summary of the characteristics of the SALURBAL cities with at least one person or child survey respondent (and thus included as part of the resource) is shown in Supplementary Table S4 (available as Supplementary data at *IJE* online). Comparedwith the entire universe of SALURBAL cities, cities with survey participants included in the resource are larger and more densely populated, and have higher socioeconomic status (SES level) (proxied by percentage of population aged 25 and older with at least primary education and percentage of households with sewage network).

## Data resource use

In general, SALURBAL health survey data analyses leverage the pooled sample of cities and countries and their linked SALURBAL geographical datasets to answer specific research questions (while accounting for the multi-level nature of data). In some circumstances, country-specific SALURBAL health survey data have been used to answer research questions that rely on linkage to country-specific datasets (e.g. Mexico’s Retail Food Environment dataset).

The types of research questions related to urban health that can be investigated with this resource include: (i) between-city differences (e.g. how much does individual-level health vary across cities, what urban features are related to this variability); (ii) within-city differences (e.g. what sub-city or neighbourhood level attributes on levels are related to health outcomes and risk factors); (iii) urban social inequalities in risk factors or health outcomes and city factors associated with larger or smaller inequalities (e.g. effect modification by city characteristics); and (iv) changes over time (e.g. are changes over time in city or sub-city characteristics related to changes in individual-level health outcomes or inequalities).

To date, the data resource has been used extensively. A list of all the publications can be found in the SALURBAL Portal: [https://data.lacurbanhealth.org]. Examples of completed studies include: association of education level with diabetes prevalence in Latin American cities and its modification by city characteristics^6^; associations of urban environment features with hypertension,^7^ diabetes and obesity,^8^ depressive symptoms^9^; associations between social environment with self-rated health,^10^ non-communicable diseases;^11^^,^^12^ associations of city-level women’s empowerment and income inequality with excess weight^13^; longitudinal changes in the retail food environment in Mexico and their association with diabetes^14^ and hypertension^15^; and racial disparities in self-rated health across Brazilian cities and segregation.^16^

## Strengths and weaknesses

The SALURBAL health survey data resource represents a novel and unique resource that allows the characterization and study of important within- and between-city differences as well as inequities in self-reported health and health risks factors across a large set of cities in different countries in Latin America. A major strength of the SALURBAL data resource is the integrated, multi-level structure that allows for flexibility in spatial and temporal scales depending on the research question and data available, and the possibility of different analytical approaches (e.g. ecological analyses, small-area analyses, multi-level analyses). The sample includes a very large, highly diverse population with respect to age, socioeconomic background and health status, living in highly diverse contexts, including social and economic factors, built and natural environments and other environmental exposures. Other strengths of the SALURBAL health survey data resource include its potential for expansion to include other health outcomes and additional linkages and extension to other time periods. SALURBAL represents a model for cross-country collaboration and data sharing that promotes transdisciplinary work.

Limitations of the data include: (i) the lack of recent health survey data in some countries (e.g. Central America); (ii) the challenges of retrospective data harmonization, as described in the data production section, which forced us to be focused and intentional but limited the domains we could handle; (iii) heterogeneity in the availability and quality of the data across countries, with many important health risk factors not being universally assessed in the health surveys of the region (e.g. mental health-related outcomes, violence, health care access and use) and with limited coverage across urban areas (e.g. Brazil); (iv) the almost exclusive reliance on self- reported outcomes (with the exception of height and weight and blood pressure in some countries and some subsamples); (v) the surveys were not designed to be representative of the SALURBAL cities and survey design features and weights cannot be used; although we have developed approaches to derive city-level prevalences, these are imperfect and only approximations; (vi) by design, survey respondents represent people living in households.

Beyond the resource itself, our experience with the creation of this data resource adds to the literature around data pooling and harmonization efforts.^17–22^ We summarize our top 10 recommendations in handling and harmonizing health survey data in Box 1.

## Data resource access

Following country-specific reviews and necessary approvals and to facilitate and expedite data harmonization and sharing, all survey datasets have been archived by the Drexel Data and Methods Core. Metadata describing the harmonized measures available in the SALURBAL study are found on the SALURBAL Data Portal [https://data.lacurbanhealth.org]. Access to SALURBAL datasets is granted to investigators with manuscript proposals approved by SALURBAL. Limited SALURBAL datasets, without personal identifiers such as addresses, and other restricted-use data will be released for use in accordance with country-specific or other agreements. Some aggregated indicators are available publicly on the SALURBAL Data Portal. Queries about using the data can be sent to [salurbal.data@drexel.edu]. The resource will be maintained at least through 2028 under SALURBAL-Climate grant funded by the Wellcome Trust.

## Ethics approval

The SALURBAL study protocol was approved by the Drexel University Institutional Review Board (IRB) with ID #1612005035 and by appropriate site-specific IRBs. Data use agreements are made with country agencies specific to each survey, given what data can be shared or made public and under what conditions.

## Supplementary Material

dyae171_Supplementary_Data

## Data Availability

See ‘Data Resource Access’ above.
