# Supplementary material for: Data Resource Profile: Harmonized health survey data for 240 cities across 11 countries in Latin America: the SALURBAL project
Source: Int J Epidemiol. 2025 Jan 6;54(1):dyae171. doi: 10.1093/ije/dyae171 (PMC11703366; doi:10.1093/ije/dyae171)
Supplement: dyae171_Supplementary_Data [file dyae171_supplementary_data.docx]

Supplementary material

Table of Contents

[Supplementary Figure S1. Salud Urbana en America Latina/Urban Health in Latin America (SALURBAL) cities* with and without survey data. 3](#_Toc182993193)

[Supplementary Table S1: Characteristics of health surveys included in Salud Urbana en America Latina/Urban Health in Latin America (SALURBAL) data resource 5](#_Toc182993194)

[Supplementary Figure S2. Number of surveys, cities, and participants: Salud Urbana en America Latina/Urban Health in Latin America (SALURBAL) health survey component. 13](#_Toc182993195)

[Supplementary Figure S3: Salud Urbana en America Latina/Urban Health in Latin America (SALURBAL) Health Survey Data resource inclusion criteria 15](#_Toc182993196)

[Supplementary Figure S4: Salud Urbana en America Latina/Urban Health in Latin America (SALURBAL)Health Survey Data Harmonization workflow 16](#_Toc182993197)

[Supplementary Table S2: Harmonized variables included in Salud Urbana en America Latina/Urban Health in Latin America (SALURBAL) data resource and harmonization characteristics 17](#_Toc182993198)

[Supplementary Table S3: Availability of harmonized variables included in Salud Urbana en America Latina/Urban Health in Latin America (SALURBAL) data 38](#_Toc182993199)

[Supplementary Figure S5: Estimated prevalence of overweight for Salud Urbana en America Latina/Urban Health in Latin America (SALURBAL) cities and countries, by sex. (standardized to the pooled 2010 SALURBAL population age distribution) 46](#_Toc182993200)

[Supplementary Figure S6: Estimated prevalence of diabetes for Salud Urbana en America Latina/Urban Health in Latin America (SALURBAL) cities and countries, by sex. (standardized to the pooled 2010 SALURBAL population age distribution) 47](#_Toc182993201)

[Supplementary Figure S7: Estimated prevalence of hypertension for Salud Urbana en America Latina/Urban Health in Latin America (SALURBAL) cities and countries, by sex. (standardized to the pooled 2010 SALURBAL population age distribution) 48](#_Toc182993202)

[Supplementary Figure S8: Estimated prevalence of poor-fair self reported health for Salud Urbana en America Latina/Urban Health in Latin America (SALURBAL) cities and countries, by sex. (standardized to the pooled 2010 SALURBAL population age distribution) 49](#_Toc182993203)

[Supplementary Figure S9: Estimated prevalence of smoking for Salud Urbana en America Latina/Urban Health in Latin America (SALURBAL) cities and countries, by sex. (standardized to the pooled 2010 SALURBAL population age distribution) 50](#_Toc182993204)

[Supplementary Figure S10: Estimated prevalence of adult (20 years and older) obesity over time in 46 selected Salud Urbana en America Latina/Urban Health in Latin America (SALURBAL) Mexican cities, by sex (standardized to the Mexico population age distribution). 51](#_Toc182993205)

[Supplementary table S4. Selected characteristics of SALURBAL cities. All Salud Urbana en America Latina/Urban Health in Latin America (SALURBAL) cities and SALURBAL cities with at least one survey available and included in this resource. 52](#_Toc182993206)

[Methods for city-level prevalence estimates 54](#_Toc182993207)

[References 65](#_Toc182993208)

# Supplementary Figure S1. Salud Urbana en America Latina/Urban Health in Latin America (SALURBAL) cities* with and without survey data.


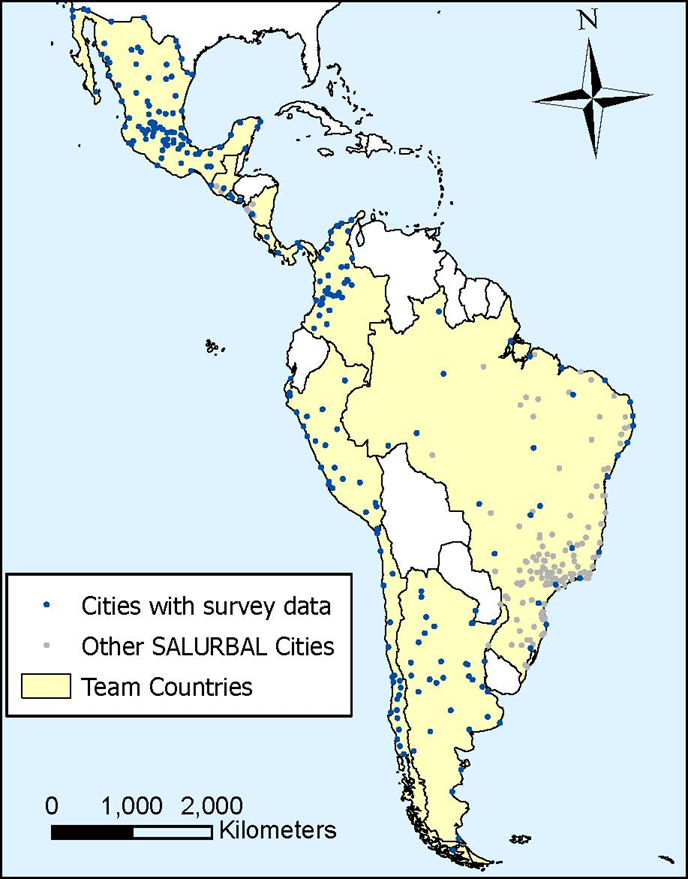


Adapted from Fig. 2 in Quistberg DA, Diez Roux AV, Bilal U, et al. Building a Data Platform for Cross-Country Urban Health Studies: the SALURBAL Study. Journal of urban health : bulletin of the New York Academy of Medicine 2019;96:311-37.

* See: <https://drexel-uhc.github.io/salurbal-city-selection-scrolly/> for definition and selection of SALURBAL city, sub-city and neighborhood.

Supplementary Table S1: Characteristics of health surveys included in Salud Urbana en America Latina/Urban Health in Latin America (SALURBAL) data resource

| Country | Survey/Years | Sample Characteristics | Includes Child data | Sampling Strategy | Geographic coverage | Over-  sampling | | Representation |
| --- | --- | --- | --- | --- | --- | --- | --- | --- |
| Argentina | Encuesta Nacional de Factores de Riesgo, ENFR (National Risk Factors Survey)  Years: 2005, 2009, 2013 | Age: >18 years  Total N: 41,392 [2005], 34,732 [2009], 32,365 [2013]  N in SALURBAL: 25,753 [2005], 16,218 [2009], 21,451 [2013] | No | Multistage [Aglomerado censal; área (groups of radio censales); household; person 18 years or older]  Stratified [population size; education level of head of household] | *Localidades* with over 5,000 population | None | | National, four *localidades* groups based on size, 6 regions, 23 provinces, Ciudad Autonoma de Buenos Aires, and 8 metropolitan areas >500,000 population. |
| Brasil | Pesquisa Nacional de Saúde, PNS (National Health Survey)  Years: 2013, 2019 | Age: All ages [2013], adults 18+ years [2019]  Total N: 64,308 adults 18+ years [2013], 91,285 adults 18+ years [2019]  N in SALURBAL: 29,353 in L2s; 40,703 in L1ADs adults 18+ years [2013], 33,515 in L2s; 46,767 in L1ADs adults 18+ years [2019] | No | Multistage [census tracts or groups of census tracts; households; person 18 years or older]  Stratified [capital city, metropolitan region, or integrated economic development region, then rest of municipalities; Urban/rural; total household income] | Regions (5) States or federation units (27), state capitals (27) | None | | Regions (5) States or federation units (27), state capitals (27), urban and rural, metropolitan areas and development integrated areas |
| Chile | Encuesta Nacional de Salud, ENS (National Health Survey)  Years: 2003, 2010, 2017 | Age: ≥15 years [2003], ≥15 years [2010, 2017]  Total N: 3,583 [2003], 5,293 [2010], 6,233 [2017]  N in SALURBAL: 2,032 [2003], 3,140 [2010], 3,805 [2017] | No | Multistage [Comunas; Segments within comunas; household; person 17 [2003] or 15 [2010, 2017] years or older]  Stratified [urban/rural with three groups of population sizes] | National | Adults ≥65, regions distinct to Metropolitan Region, rural areas | | National, Regions (15), urban/rural |
| Chile | Country: Chile  Survey: Encuesta Longitudinal de Primera Infancia (ELPI) (Longitudinal Survey of Early Childhood) | Age: 1-12 years  Total N: 17,307 children 1-12 years  N in SALURBAL: 6,723  Year: 2017-2018 | Yes | Stratified multistage sampling.  First stage: Strata of comunas with similar socioeconomic status. Second stage: systematic random sampling  One child per household was selected. | National | None | | National  Urban/Rural |
| Colombia | Encuesta Nacional de Salud, ENS (National Health Survey)  Year: 2007 | Age: 0 – 69 years  Total N: 102,677 (41,281 adults 18-69 years)  N in SALURBAL: 43,182 | No | Multistage [Municipalities or combination of municipalities if small; Manzanas; household; person adults 18-69 and all children 17 and under]  Stratified [region; urbanization of municipal seats; urban/rural municipal population; unsatisfied basic needs] | National | None | | Region, department, subregion, urban area of municipal capitals, urban/rural, by poverty level |
| Colombia | Encuesta Nacional de la Situation Nutricional en Colombia, ENSIN (National Nutritional Situation in Colombia)  Year: 2005, 2010, 2015 | Age: 0-69 years  Total N: 92,357 adults 18-69 years [2005], 115,437 adults 18-69 years [2010], 87,819 adults 18-69 years [2015]  N in SALURBAL: 42,336 adults 18-69 years [2005], 55,863 adults 18-69 years [2010], 36,593 adults 18-69 years [2015]  Total N: 61,836 children <18 years [2005], 75,470 children <18 years [2010], 49,901 children <18 years [2015]  N in SALURBAL: 23,794 children <18 years [2005], 30,278 children <18 years [2010], 17,104 children <18 years [2015] | Yes | Multistage [Municipalities or combination of municipalities if small; Rural blocks or sections; household segments; person adults 18-69 and children 17 and under]  Stratified [region; urbanization of municipal seats; urban/rural municipal population; unsatisfied basic needs] | National | None | | National, regional, departmental (anthropometry)  National, regional (physical activity) |
| Costa Rica | Encuesta Multinacional de Diabetes mellitus y Factores de Riesgo, CAMDI (Multinational Survey of Diabetes Mellitus & Risk Factors, Central American Diabetes Initiative)  Year: 2005 | Age: ≥20 years  Total N: 1,427  N in SALURBAL: 1,427 | No | Multistage [Census segments; groups of households (compactos); Persons within three age groups (1 selected from 20-39 years, 1 selected from 40-64 years, all selected from ≥65 years)] | Metropolitan San Jose | Age ≥ 65 | | Metropolitan San Jose |
| Guatemala | Encuesta Multinacional de Diabetes mellitus y Factores de Riesgo, CAMDI (Multinational Survey of Diabetes Mellitus & Risk Factors, Central American Diabetes Initiative)  Year: 2005 Year: 2002-2003 | Age: ≥20 years  Total N: 1,397  N in SALURBAL: 1,397 | No | Multistage [Segmento censal, groups of dwellings (compacto); all household members 20 years and older] | Villa Nueva Municipio, a part of metropolitan Guatemala City | None | | Villa Nueva Municipio |
| Guatemala | Demographic and Health Survey (DHS) Year: 2014-2015 | Age: 0-4 (males and females) and 15-49 (females only)  Total N: 23,343 females 18-49  N in SALURBAL: 2,730 females 18-49 years  Total N: 12,851 children <5 years  N in SALURBAL: 983 children <5 years | Yes | A multistage, random, stratified cluster-sampling procedure  was used to select a nationally representative sample | National | None | | National, Department |
| Nicaragua | Encuesta Multinacional de Diabetes mellitus y Factores de Riesgo, CAMDI (Multinational Survey of Diabetes Mellitus & Risk Factors, Central American Diabetes Initiative)  Year: 2005 Year: 2003 | Age: ≥20 years  Total N: 1,993  N in SALURBAL: 1,397 | No | Multistage [Urban districts divided into 50 strata, groups of households (compacto); all family members living together 20 years and older] | Municipality of Managua | None | | Municipality of Managua |
| Mexico | Encuesta National de Salud, ENSA (National Health Survey) Year: 2000 | Age: all ages  Total N: 48,836 adults 18+years  N in SALURBAL: 29,733 adults 18+years  Total N: 42,042 children <18 years  N in SALURBAL: 23,966 children <5 years | Yes | Multistage [AGEB; Manzana (urban) or pseudo-manzanas withig localidades (rural); Households; 1 person within each of the groups (0-4 years, 5-9 years, 10-19 years, 20 years and older, recent medical service user)] | National | None | | National, state, metropolitan areas |
| Mexico | Encuesta Nacional de Salud y Nutricion, ENSANUT (National Survey for Health and Nutrition) Years: 2006, 2012, 2016, 2018 | Age: all ages  Total N: 55,108 adults 18+ years [2006], 46,277 adults 18+ years [2012], 29,797 adults 18+ years [2016], 44,514 adults 18+ years [2018]  N in SALURBAL: 31,532 adults 18+ years [2006], 26,335 adults 18+ years [2012], 14,618 adults 18+ years [2016], 27,118 adults 18+ years [2018]  Total N: 34,752 children <18 years [2006],45,757 children <18 years [2012], 7,308 children <18 years [2016], 15,019 children <18 years [2018]  N in SALURBAL: 19,431 children <18 years [2006], 25,014 children <18 years [2012], 3,274 children <18 years [2016], 7,538 children <18 years [2018] | Yes | Multistage [AGEB; Manzana (urban) or pseudo-manzanas withig localidades (rural); Households; 1 person within each of the groups (0-4 years, 5-9 years, 10-19 years, 20 years and older, recent medical service user)]  Stratified [socioeconomic status of AGEB at the state level] | National | AGEB with the highest index of poor socioeconomic conditions^b^ | | National, state, metropolitan areas, urban/rural, high/low socioeconomic status |
| Panama | Encuesta Nacional de Salud y Calidad de Vida ENSCAVI (National Survey of Health and Quality of Life) Years: 2007 | Age: ≥18 years  Total N: 25,748  N in SALURBAL: 11,394 | No | Multistage [Census segments; Dwellings; Persons ≥18 years]  Stratified [Indigenous population in province; Urban/rural] | National | None | | National, District |
| Peru | Encuesta Nacional de Demografia y Salud, ENDES (National Survey of Demographics and Health) Year: 2016 | Age: All ages  Total N: 122,368 (adults 18+ years N=32158)  N in SALURBAL: 11,929 adults 18+ years  Total N: 22,682 children <5 years, 1914 children 15-17 years;  N in SALURBAL: 8,547 children <5 years, 668 children 15-17 years.  Children aged 5-14 were not included. | Yes | Multistage [Conglomerado (set of census blocks – urban) or Empadronamiento (set of households – rural); Households; One person within each of the groups (>15 years, females 15-49 years, children <5 years, children <12 years)]  Stratified [Department; Urban/Rural] | National | None | | National, Urban National, Rural National, Natural Region: Lima Metropolitan area, coast/mountain/jungle |
| El Salvador | Encuesta Multinacional de Diabetes mellitus y Factores de Riesgo, CAMDI (Multinational Survey of Diabetes Mellitus & Risk Factors, Central American Diabetes Initiative)  Year: 2005 Year: 2004 | Age: ≥20 years  Total N: 1,872  N in SALURBAL: 1,872 | No | Multistage [Segmento censal, groups of dwellings (compacto); all household members 20 years and older]^a^ | Municipio of Santa Tecla | Unknown | | Municipio of Santa Tecla |
| El Salvador | Encuesta Nacional de Salud Familiar (National Family Health Survey)  Year: 2008 | Age: 0-4 (males and females) and 15-49 (females only)  Total N: 12,091 females 18-49  N in SALURBAL: 4,297 females 18-49 years  Total N: 4,637 children <5 years; 1,218 females 15-17  N in SALURBAL: 1,290 children <5 years; 338 females 15-17 | Yes | A multistage, random, stratified cluster-sampling procedure  was used to select a nationally representative sample | National | Department of San Salvador | | National, Department |
| El Salvador | Encuesta Nacional de Enfermedades Cronicas no transmisibles en Poblacion Adulta de El Salvador ENECA (National Survey of Noncommunicable Chronic Diseases in the Adult Population of El Salvador) Year: 2014-2015 | Age: ≥20 years  Total N: 4,817  N in SALURBAL: 1,546 | No | Two-stage [Segmento censal, groups of dwellings (compacto); all household members 20 years and older] | National | None | | National, Urban National, Rural |
| AGEB: Area Geoestadistica Basica  CAMDI: Encuesta Multinacional de Diabetes mellitus y Factores de Riesgo (Multinational Survey of Diabetes Mellitus & Risk Factors, Central American Diabetes Initiative)  DHS: Demographic and Health Survey  ELPI: Encuesta Longitudinal de Primera Infancia (Longitudinal Survey of Early Childhood)  ENDES: Encuesta Nacional de Demografia y Salud (National Survey of Demographics and Health)  ENECA: Encuesta Nacional de Enfermedades Cronicas no transmisibles en Poblacion Adulta de El Salvador (National Survey of Noncommunicable Chronic Diseases in the Adult Population of El Salvador)  ENFR: Encuesta Nacional de Factores de Riesgo (National Risk Factors Survey)  ENS: Encuesta Nacional de Salud (National Health Survey)  ENSA: Encuesta National de Salud (National Health Survey)  ENSANUT: Encuesta Nacional de Salud y Nutricion (National Survey for Health and Nutrition)  ENSCAVI: Encuesta Nacional de Salud y Calidad de Vida (National Survey of Health and Quality of Life)  ENSIN: Encuesta Nacional de la Situation Nutricional en Colombia (National Nutritional Situation in Colombia)  FESAL: Encuesta Nacional de Salud Familiar (National Family Health Survey)  PNS: Pesquisa Nacional de Saúde (National Health Survey)  ^a^ Documentation for El Salvador’s survey design is based on the design of other countries in the CAMDI project.  ^b^ In Mexico, The households with the greatest deficiencies were identified through the construction of a defined social lag(rezago) index for the AGEBs; the index that was built is similar to the social lag (rezago) index built by the National Evaluation Council of the Social Development Policy for localities in 2005. <https://www.coneval.org.mx/rw/resource/coneval/med_pobreza/1024.pdf> | | | | | | |

# Supplementary Figure S2. Number of surveys, cities, and participants: Salud Urbana en America Latina/Urban Health in Latin America (SALURBAL) health survey component.

**
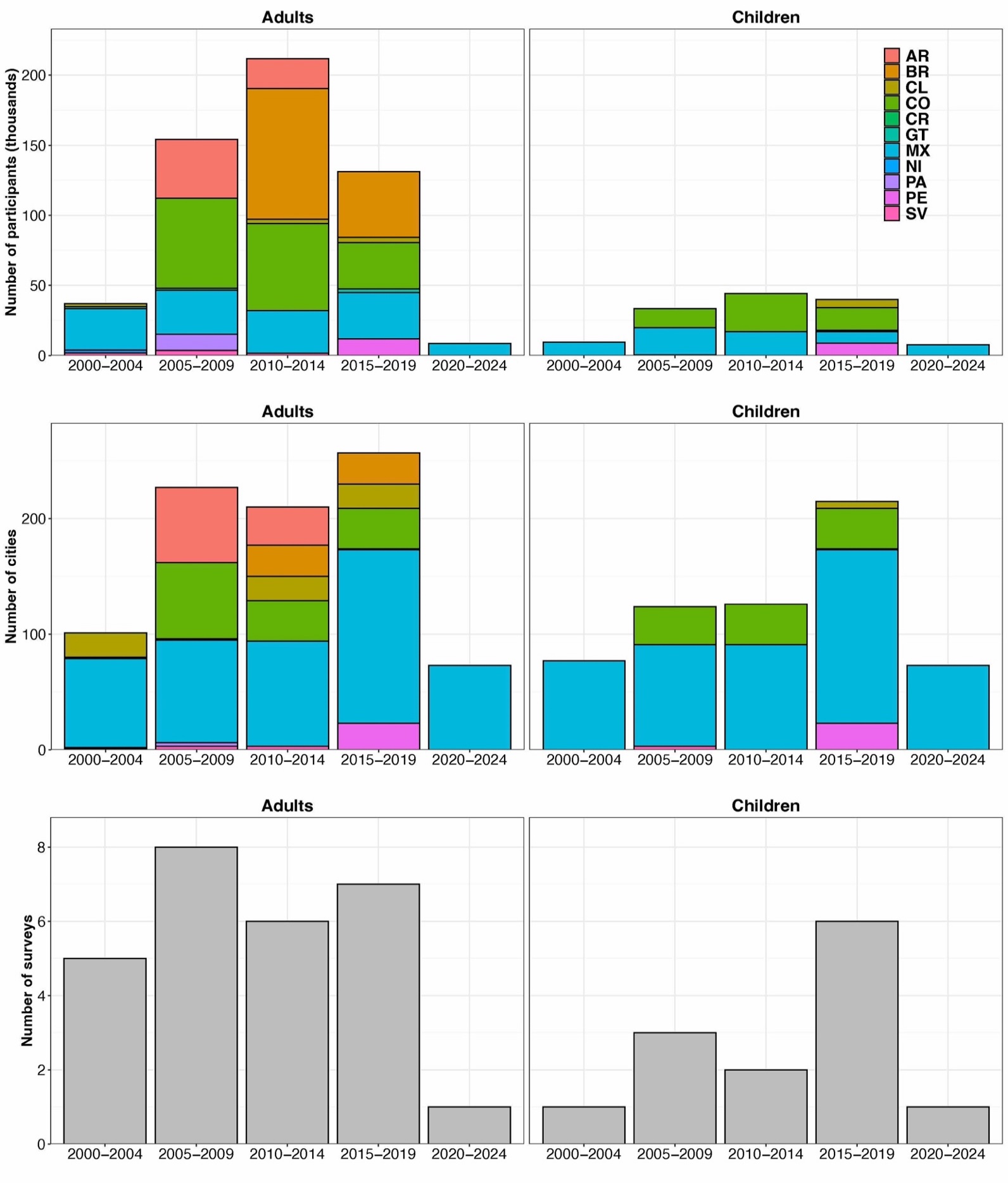
**

AR: Argentina, BR: Brazil, CL: Chile, CO: Colombia, CR: Costa Rica, GT: Guatemala, MX: Mexico, NI: Nicaragua, PA: Panama, PE: Peru, SV: El Salvador

# Supplementary Figure S3: Salud Urbana en America Latina/Urban Health in Latin America (SALURBAL) Health Survey Data resource inclusion criteria

# Supplementary Figure S4: Salud Urbana en America Latina/Urban Health in Latin America (SALURBAL)Health Survey Data Harmonization workflow

# Supplementary Table S2: Harmonized variables included in Salud Urbana en America Latina/Urban Health in Latin America (SALURBAL) data resource and harmonization characteristics

| **Domain** | **Variable Name** | **Description** | **Response Options** | **Question Scenarios%** | **Harmonization type$** | **Notes** | **Reference** |
| --- | --- | --- | --- | --- | --- | --- | --- |
| Demographics | SVYMALE | Sex of respondent | Female Male | 1 | Type 1 |  |  |
| Demographics | SVYAGE | Age in years | Numeric in years | 2 | Type 2 |  |  |
| Demographics | SVYAGEMO | Age in months | Numeric in months | 2 | Type 2 |  |  |
| Demographics | SVYMARSTAT | Marital Status | Married/partnership Divorced/Separated Widowed Single/Never married | 2 | Type 2 |  |  |
| Demographics | SVYAFRODEC | Afro-decent as characterized by self-reported race. | Yes, Afro-decent No | Each survey where available was different | Type 2 |  |  |
| Demographics | SVYINDIGENOUS | Indigenous self-identification | Yes, Indigenous No | Each survey where available was different | Type 2 |  |  |
| Diabetes | SVYDBDX1 | Diabetes mellitus diagnosis Version 1. A respondent is classified as "yes" if they report having been told by a physician or health care provider that they have diabetes or high blood sugar levels. Women who report that a diabetes diagnosis was made during pregnancy are classified as NOT having diabetes. | Yes No | 8 | Type 3 | Not all surveys asked specifically about gestational diabetes. Because of this, we created two versions of diabetes with and without gestational diabetes. If gestational diabetes could not be separated, then we assumed that women who had gestational diabetes answered "yes" |  |
| Diabetes | SVYDBDX2 | Diabetes mellitus diagnosis Version 2. A respondent is classified as "yes" if they report having been told by a physician or health care provider that they have diabetes or high blood sugar levels including women who report that a diabetes diagnosis was made during pregnancy. | Yes No | 8 | Type 3 |  |  |
| Diabetes | SVYDBMED | Current Pharmacological Treatment among diabetics. A diabetic is defined as receiving any pharmacological treatment if they report using insulin or medications “to lower sugar” or control diabetes prescribed by a health care provider. Those not receiving either of these treatments are considered to not be receiving any pharmacological treatment. | Yes No | Each survey where available was different | Type 2 |  |  |
| Hypertension | SVYHTDX1 | Hypertension diagnosis Version 1. Presence of hypertension diagnosis by a health care provider among all adults, where adult female respondents who report a hypertension diagnosis during pregnancy are considered to be non-hypertensive. A survey participant is classified as having hypertension if they report having been told by a physician or health care provider that they have high blood pressure or hypertension | Yes No | Each survey where available was different | Type 3 | Not all surveys asked specifically about gestational hypertension. Because of this, we created two versions of self-reported hypertension with and without gestational hypertension. If gestational hypertension could not be separated, then we assumed that women who had gestational hypertension answered "yes" |  |
| Hypertension | SVYHTDX2 | Hypertension diagnosis Version 2. Presence of hypertension diagnosis by a health care provider among adults regardless of whether hypertension was diagnosed during pregnancy or not (adult female respondents who report a hypertension diagnosis during pregnancy are considered to be hypertensive). A survey participant is classified as having hypertension (hypertension or hypertension during pregnancy) if they report having been told by a physician or health care provider that they have hypertension or high blood pressure. | Yes No | Each survey where available was different | Type 3 |  |  |
| Hypertension | SVYHTMED | Current Pharmacological Treatment for hypertension (Hypertension Version 3). A participant is defined as hypertensive if they report that a physician told them that they had hypertension AND if they report using medications “to lower blood pressure” or control hypertension prescribed by a health care provider. | Yes No | Each survey where available was different | Type 2 |  |  |
| Hypertension | SVYHTMBP | Hypertension Version 4. Hypertension status as defined by having hypertension medication use OR Systolic Blood Pressure>=140 OR Diastolic Blood Pressure>=90. | Yes No | Each survey where available was different | Type 2 |  | U.S. Centers for Disease Control National Health and Nutrition Examination Survey (NHANES) (https://www.ahajournals.org/doi/pdf/10.1161/CIRCOUTCOMES.111.963439) |
| Hypertension | SVYHTCTRL | Hypertension control. Hypertension control is defined using NHANES recommendation as persons currently using hypertension medication AND systolic blood pressure<140 AND diastolic blood pressure<90. Uncontrolled hypertension is defined as persons currently using hypertension medication AND [systolic blood pressure ≥ 140 OR diastolic blood pressure ≥ 90]. Hypertension control is only defined amongst respondents who answered “yes” to using hypertension medications. | Controlled Not controlled | Each survey where available was different | Type 2 |  |  |
| Hypertension | SVYSBP | Systolic blood pressure as the average of all systolic blood pressure measurements available. | Numeric | 3 | Type 2 | The number of blood pressure measurements taken ranged between 1-3. If there were more than 1 measurements, most surveys required 5 minutes between measurements. |  |
| Hypertension | SVYDBP | Diastolic blood pressure as the average of all diastolic blood pressure measurements available. | Numeric | 3 | Type 2 |  |  |
| Anthropometry | SVYWTKG | Weight in kilograms as measured by the interviewer. If more than one measurement was taken, this is the average of all available measurements. | Numeric | 2 | Type 2 | The number of weight measurements taken ranged between 1-2. |  |
| Anthropometry | SVYHTCM | Height in centimeters as measured by the interviewer. If more than one measurement was taken, this is the average of all available measurements. | Numeric | 2 | Type 2 |  |  |
| Anthropometry | SVYBMI | Body mass index in kg/m2 calculated from measured height and weight. | Numeric | 1 | Type 1 |  | World Health Organization (WHO) (http://apps.who.int/bmi/index.jsp?introPage=intro_3.html) |
| Anthropometry | SVYBMIZ | Body Mass Index (BMI)-for-age z-score. This measure is calculated from the child’s weight and length/height. This measure is especially useful for screening for overweight and obesity. This measure is available for all children aged 0-17 but recommended for use only in children aged 5-17. | Numeric | 1 | Type 1 |  | World Health Organization (WHO) (https://www.who.int/childgrowth/training/module_c_interpreting_indicators.pdf) |
| Anthropometry | SVYHTAGZ | Height for age z-score. This measure reflects the attained growth in length or height at the child’s age. This indicator can identify children with stunted growth. | Numeric | 1 | Type 1 |  |  |
| Anthropometry | SVYWTHTZ | Weight for height z-score. This measure reflects body weight in proportion to attained growth in length or height. This measure can be used to identify children with low weight-for-height who may be wasted or severely wasted as well as identify children with high weight-for-length/height who may be at risk of becoming overweight or obese. This measure is available and recommended for children aged 0-60 months. It is not calculated for children aged 5-17. | Numeric | 1 | Type 1 |  |  |
| Anthropometry | SVYWTAGZ | Weight for age z-score. This measure reflects body weight relative to the child’s age. This indicator can identify whether a child is underweight or severely underweight, but it is not used to classify a child as overweight or obese. This measure is available for all children aged 0-120 months. | Numeric | 1 | Type 1 |  |  |
| Anthropometry | SVYGRTHST | Growth standard category. Based on BMI Z-score for those over 60 months and weight for height Z-score for those 60 months and younger. Severe underweight only defined in those 60 months and younger. | Severe underweight Underweight Normal Overweight Obese | 1 | Type 1 |  |  |
| Anthropometry | SVYWTKGSELF | Self-reported weight in kilograms. | Numeric | 2 | Type 2 |  |  |
| Anthropometry | SVYHTCMSELF | Self-reported height in centimeters. | Numeric | 2 | Type 2 |  |  |
| Anthropometry | SVYBMISELF | Body mass index in kg/m2 calculated from self-reported height and weight. | Numeric | 1 | Type 1 |  | World Health Organization (WHO) (http://apps.who.int/bmi/index.jsp?introPage=intro_3.html) |
| Health Status | SVYHLTHSTAT1 | Self-rated Health status Version 1 with response rating of poor to excellent | Excellent Very good Good Fair Poor | 1 | Type 3 |  |  |
| Health Status | SVYHLTHSTAT2 | Self-rated Health status Version 2 with response rating of very poor to very good | Very good Good Fair Poor Very poor | 1 | Type 3 |  |  |
| Health Status | SVYHLTHSTAT3 | Self-rated Health status Version 3 with response rating of very unsatisfied to very satisfied | Very satisfied Satisfied Unsatisfied Very unsatisfied | 1 | Type 3 |  |  |
| Tobacco Use | SVYCIGHX1 | Lifetime cigarette use (100+ or 5 packs). Respondents are classified as never, former or current smokers of at least 100 cigarettes or 5 packs of cigarettes. | Never Former Current | Each survey where available was different | Type 3 | Due to differences in the way questions about smoking status were asked in each survey, four versions of each lifetime and current smoking variable were created depending on whether questions specified cigarette use or only tobacco use (without specifying cigarettes). The number of cigarette or tobacco products (such as 100 or more or 5 packs) is not consistent across surveys. | United States Center for Disease Control (https://www.cdc.gov/nchs/nhis/tobacco/tobacco_glossary.htm#:~:text=Every%20day%20smoker%3A%20An%20adult,at%20the%20time%20of%20interview.) |
| Tobacco Use | SVYCIGHX2 | Lifetime cigarette use (amount not specified). Respondents are classified as never, former or current smokers (the number of cigarettes is not defined). | Never Former Current | Each survey where available was different | Type 3 |  |  |
| Tobacco Use | SVYTOBHX1 | Lifetime smoked tobacco use (100+). Respondents are classified as never, former or current smokers of at least 100 or more ciga-rettes/cigars/pipes. | Never Former Current | Each survey where available was different | Type 3 |  |  |
| Tobacco Use | SVYTOBHX2 | Lifetime smoked tobacco use (amount not specified). Respondents are classified as never, former or current smokers (the number of cigarettes/cigars/pipes is not defined). | Never Former Current | Each survey where available was different | Type 3 |  | World Health Organization (https://www.who.int/data/gho/data/indicators/indicator-details/GHO/gho-tobacco-control-monitor-current-tobaccouse-tobaccosmoking-cigarrettesmoking-agestd-tobagestdcurr) |
| Tobacco Use | SVYCIGCUR1 | Current cigarette use (100+ or 5 packs). Respondents are classified as yes or no current smokers amongst those who ever smoked of at least 100 cigarettes or 5 packs of cigarettes. | Yes, Current smoker No | Each survey where available was different | Type 3 |  | United States Center for Disease Control (https://www.cdc.gov/nchs/nhis/tobacco/tobacco_glossary.htm#:~:text=Every%20day%20smoker%3A%20An%20adult,at%20the%20time%20of%20interview.) |
| Tobacco Use | SVYCIGCUR2 | Current cigarette use (amount not specified). Respondents are classified as yes or no current smokers (the number of cigarettes is not defined). | Yes, Current smoker No | Each survey where available was different | Type 3 |  |  |
| Tobacco Use | SVYTOBCUR1 | Current smoked tobacco use (100+). Respondents are classified as yes or no current smokers amongst those who smoked at least 100 or more ciga-rettes/cigars/pipes during their lifetime. | Yes, Current smoker No | Each survey where available was different | Type 3 |  |  |
| Tobacco Use | SVYTOBCUR2 | Current smoked tobacco use (amount not specified). Respondents are classified as yes or no current smokers (the number of cigarettes/cigars/pipes is not defined). | Yes, Current smoker No | Each survey where available was different | Type 3 |  | World Health Organization (https://www.who.int/data/gho/data/indicators/indicator-details/GHO/gho-tobacco-control-monitor-current-tobaccouse-tobaccosmoking-cigarrettesmoking-agestd-tobagestdcurr) |
| Alcohol Use | SVYCURRALC1 | Current alcohol use Version 1 defined as having consumed at least one alcoholic beverage within last 30 days. | Yes No | Each survey where available was different | Type 3 | Some surveys asked "last 30 days" while others asked "past month." We considered these to both be equivalent. |  |
| Alcohol Use | SVYCURRALC2 | Current alcohol use Version 2 defined as having consumed at least one alcoholic beverage within last 12 months. | Yes No | Each survey where available was different | Type 3 | Some surveys asked "last 12 months" while others asked "past year." We considered these to both be equivalent. | United States Center for Disease Control (CDC) (https://www.cdc.gov/alcohol/faqs.htm) |
| Alcohol Use | SVYBINGE1 | Binge drinking Version 1 defined as drinking 4 or more drinks for female, 5 or more drinks for males in last 30 days | Yes No | Each survey where available was different | Type 3 | Some surveys asked "last 30 days" while others asked "past month." We considered these to both be equivalent. | World Health Organization (WHO) (http://www.who.int/gho/alcohol/consumption_patterns/heavy_episodic_drinkers_text/en/) |
| Alcohol Use | SVYBINGE2 | Binge drinking Version 2 defined as drinking 5 or more drinks for both sexes in last 30 days | Yes No | Each survey where available was different | Type 3 | Some surveys asked "last 30 days" while others asked "past month." We considered these to both be equivalent. |  |
| Alcohol Use | SVYBINGE3 | Binge drinking Version 3 defined as drinking 5 or more drinks for both sexes in last 15 days | Yes No | Each survey where available was different | Type 3 |  |  |
| Alcohol Use | SVYBINGE4 | Binge drinking Version 4 defined as drinking 3 or more drinks for female, 4 or more drinks for males in last 30 days | Yes No | Each survey where available was different | Type 3 | Some surveys asked "last 30 days" while others asked "past month." We considered these to both be equivalent. |  |
| Pregnancy | SVYEVERPREG | Ever pregnant indicates whether adult female respondents indicate that they have ever been pregnant. Pregnancies include all pregnancies including pregnancies that did not come to full term such as premature birth, miscarriage, stillborn, and/or abortion. | Yes No | 4 | Type 2 | Some surveys asked specifically if the respondent had ever been pregnant while other asked about the total number of pregnancies in the lifetime. If total number of pregnancies was >0, we assigned as "yes" | . |
| Pregnancy | SVYCURRPREG | Currently pregnant indicates whether or not an adult female respondent indicates that she is currently pregnant at the time of the survey. | Yes No | 1 | Type 1 |  |  |
| Socioeconomic Status | SVYEDU | Highest Education level completed by the survey respondent. | Less than Primary Primary Secondary University or higher | 8 | Type 2 | Educational level was generally obtained by a combination of last grade completed and number of years of education completed. | Integrated Public Use Microdata Series (IPUMS) (https://international.ipums.org/international-action/variables/EDATTAIN#codes_section) |
| Socioeconomic Status | SVYHHEDU | Highest Education level completed by head of household. | Less than Primary Primary Secondary University or higher | 8 | Type 2 |  |  |
| Socioeconomic Status | SVYMTHEDU | Highest Education Level completed by Mother of the child. This can be either biological or adoptive mother. | Less than Primary Primary Secondary University or higher | 3 | Type 2 |  |  |
| Socioeconomic Status | SVYFLOOR | Finished floors as defined by the main floor material is not earth, dirt, sand, or dung. | Yes, Finished No, Unfinished | 1 | Type 2 | Although the question was asked in the same way across surveys, response option differed by survey. |  |
| Socioeconomic Status | SVYWALLDUR1 | Durable walls where exterior walls are mostly composed of durable material as defined by IPUMS. IPUMS defined durable walls as manonry, stone, cement, adobe, metal, glass, and other fabricated materials. | Yes, Durable walls No, Not durable walls | 1 | Type 3 | Although the question was asked in the same way across surveys, response option differed by survey. | Integrated Public Use Microdata Series (IPUMS) (https://international.ipums.org/international-action/variables/WALL#codes_section) |
| Socioeconomic Status | SVYWALLDUR2 | Masonry walls where exterior walls are mostly composed of brick, stone, concrete, cement, and/or similar materials. | Yes, Masonry walls No, Not masonry walls | 1 | Type 3 |  |  |
| Socioeconomic Status | SVYWALLDUR3 | Durable walls as defined by country teams. | Yes, Durable walls No, Not durable walls | 1 | Type 3 |  |  |
| Socioeconomic Status | SVYNPER3RM | Overcrowding: Number of persons per room. Calculated as number of persons living in the household divided by total number of rooms excluding kitchen and bathroom | Numeric | 6 | Type 3 | The surveys were not consistent about whether total number of room or number of bedrooms was asked on the questionnaire. |  |
| Socioeconomic Status | SVYNPER25BR | Overcrowding: Number of persons per bedroom. Calculated as number of persons living in the household divided by total number of bedrooms | Numeric | 6 | Type 3 |  |  |
| Socioeconomic Status | SVYSEWANY | Sewage system of any type as defined by having a connection to a municipal sewage system, privately owned sewage system, or septic tank. | Yes No | 5 | Type 3 |  | Integrated Public Use Microdata Series (IPUMS) (https://international.ipums.org/international-action/variables/SEWAGE#codes_section) |
| Socioeconomic Status | SVYSEWNET | Sewage network connection as defined by having a public or private sewage connection. | Yes No | 5 | Type 3 |  |  |
| Socioeconomic Status | SVYWATPIPE | Piped water in the household as defined by having access to piped water on the dwelling, the property, or outside the dwelling and property as long as the water is piped. | Yes No | 7 | Type 3 |  |  |
| Socioeconomic Status | SVYWATIN | Piped water inside the dwelling as defined by having access to piped water inside the dwelling. | Yes No | 7 | Type 3 |  |  |
| Socioeconomic Status | SVYWATNET | Piped water from public network as defined by having piped water from a municipal public or private water network. | Yes No | 7 | Type 3 |  |  |
| Socioeconomic Status | SVYHHMALE | Sex of head of household. | Male Female | 2 | Type 2 | Some surveys directly asked about the sex of head of household while others identified who the head of household was on a household members inventory and sex was selected as the sex of the person identified as the head of household. |  |
| Socioeconomic Status | SVYOWNCAR | Car ownership as defined as if they or someone in their household reports owning at least one car. | Yes No | 3 | Type 2 | Some surveys asked about total number of cars owned while other surveys asked yes/no a member of the household owned a car. |  |
| Depression | SVYPHQ9A | PHQ9 Q1: Little interest or pleasure in doing things. | Not at all Several days More than half the days Nearly every day | 1 | Type 1 | Patient Health Questionnaire Depression scale | Kroenke, K., R.L. Spitzer, and J.B. Williams, The PHQ-9: validity of a brief depression severity measure. J Gen Intern Med, 2001. 16(9): p. 606-13 https://www.ncbi.nlm.nih.gov/pmc/articles/PMC1495268/ |
| Depression | SVYPHQ9B | PHQ9 Q2: Feeling down, depressed, or hopeless | Not at all Several days More than half the days Nearly every day | 1 | Type 1 |  |  |
| Depression | SVYPHQ9C | PHQ9 Q3: Trouble falling or staying asleep or sleeping too much. | Not at all Several days More than half the days Nearly every day | 1 | Type 1 |  |  |
| Depression | SVYPHQ9D | PHQ9 Q4: Feeling tired or having little energy | Not at all Several days More than half the days Nearly every day | 1 | Type 1 |  |  |
| Depression | SVYPHQ9E | PHQ9 Q5: Poor appetite or overeating | Not at all Several days More than half the days Nearly every day | 1 | Type 1 |  |  |
| Depression | SVYPHQ9F | PHQ9 Q6: Feeling bad about yourself or that you are a failure or have let your-self or your family down | Not at all Several days More than half the days Nearly every day | 1 | Type 1 |  |  |
| Depression | SVYPHQ9G | PHQ9 Q7: Trouble concentrating on things, such as reading the newspaper or watching television | Not at all Several days More than half the days Nearly every day | 1 | Type 1 |  |  |
| Depression | SVYPHQ9H | PHQ9 Q8: Moving or speaking so slowly that other people could have noticed. Or the opposite – being so fidgety or restless that you have been moving around a lot more than usual | Not at all Several days More than half the days Nearly every day | 1 | Type 1 |  |  |
| Depression | SVYPHQ9I | PHQ9 Q9: Thoughts that you would be better off dead, or of hurting yourself | Not at all Several days More than half the days Nearly every day | 1 | Type 1 |  |  |
| Depression | SVYPHQ9 | PHQ9 total score calculated as the sum of the response options for the nine questions comprising the PHQ9 questionnaire. | Numeric (ranges 0-27) | 1 | Type 1 |  |  |
| Depression | SVYCESDC | CES-D SF Q1: I felt that I could not shake off the blues, even with help from my family and friends. | Rarely or none of the time Some or little of the time Moderately or much of the time Most or almost all the time | 1 | Type 1 | Center for Epidemiological Studies - Depression scale Short form | Salinas-Rodriguez, A., et al, *Validacion de un punto de corte para la Escala de Depresion del Centro de Estudios Epidemiologicos, version abreviada (CESD-7)*. Salud Publica Mex, 2013. 55: p. 267-274 https://www.medigraphic.com/cgi-bin/new/resumen.cgi?IDARTICULO=42949 |
| Depression | SVYCESDE | CES-D SF Q2: I had trouble keeping my mind on what I was doing. | Rarely or none of the time Some or little of the time Moderately or much of the time Most or almost all the time | 1 | Type 1 |  |  |
| Depression | SVYCESDF | CES-D SF Q3: I felt depressed. | Rarely or none of the time Some or little of the time Moderately or much of the time Most or almost all the time | 1 | Type 1 |  |  |
| Depression | SVYCESDG | CES-D SF Q4: I felt that everything I did was an effort. | Rarely or none of the time Some or little of the time Moderately or much of the time Most or almost all the time | 1 | Type 1 |  |  |
| Depression | SVYCESDK | CES-D SF Q5: My sleep was restless. | Rarely or none of the time Some or little of the time Moderately or much of the time Most or almost all the time | 1 | Type 1 |  |  |
| Depression | SVYCESDP | CES-D SF Q6: I enjoyed life. | Rarely or none of the time Some or little of the time Moderately or much of the time Most or almost all the time | 1 | Type 1 |  |  |
| Depression | SVYCESDR | CES-D SF Q7: I felt sad. | Rarely or none of the time Some or little of the time Moderately or much of the time Most or almost all the time | 1 | Type 1 |  |  |
| Depression | SVYCESD7 | CESD-SF (7 questions) total score calculated as the sum of the response options for the seven questions comprising the CES-D SF questionnaire. | Numeric (Ranges 0-21) | 1 | Type 1 |  |  |
| Depression | SVYDXDEP | Doctor diagnosis of depression. A survey participant is classified as having depression if they report having been told by a physi-cian or health care provider that they have depression. | Yes No | 3 | Type 2 |  |  |
| Depression | SVYDEPTX | Receiving any treatment for depression. This includes any type of treatment including medication, psychotherapy, or other types of non-medication treatment. | Yes No | Each survey where available was different | Type 3 | Because of the way treatment is asked in each survey, medication use cannot always be separated from other types of treatment such as psychotherapy. For this reason, two versions of depression treatment were created. |  |
| Depression | SVYDEPMED | Depression medication use. A participant is defined as currently receiving any pharmacological treatment if they report using medications for depression. | Yes No | Each survey where available was different | Type 3 |  |  |
| Depression | SVYSRDEP1 | Self-reported Depressive Feelings Version 1. Defined as response to "In relation to depression/anxiety today, what best describes how you feel?" | Not anxious or depressed Moderately anxious or depressed Extremely anxious or depressed | 1 | Type 3 |  |  |
| Depression | SVYSRDEP2 | Self-reported Depressive Feelings Version 2. Defined using survey questions which directly ask the respondent “do you feel depressed” or “how often do you feel depressed” or approximate equivalent. | No days/Rarely/Never Less than half the days/Some days/Several days More than half the days/Most days/Usually Almost every day/Always/Nearly every day | Each survey where available was different | Type 3 |  |  |
| Physical Activity | SVYPALEIS | Leisure physical activity defined as moderate and vigorous physical activities carried out solely for leisure time (recreation, sport, exercise or leisure activities): Minutes per week | Numeric | 2 | Type 2 | Calculated from long IPAQ and GPAQ. | IPAQ: International Physical Activity Questionnaire. Guidelines for Data Processing and Analysis of the International Physical Activity Questionnaire. 2005. https://www.researchgate.net/file.PostFileLoader.html?id=5641f4c36143250eac8b45b7&assetKey=AS%3A294237418606593%401447163075131 World Health Organization. Global Physical Activity Questionnaire (GPAQ) Analysis Guide. 2010. https://www.who.int/ncds/surveillance/steps/resources/GPAQ_Analysis_Guide.pdf?ua=1 World Health Organization. Global recommendations on physical activity for health. Geneva: 2018. https://www.who.int/news-room/fact-sheets/detail/physical-activity#:~:text=years%20and%20above-,Should%20do%20at%20least%20150%20minutes%20of%20moderate%2Dintensity%20physical,%2D%20and%20vigorous%2Dintensity%20activity |
| Physical Activity | SVYPALEISCAT | Meeting recommendations of meeting physical activity recommendations via leisure physical activity. Meeting recommendations is defined as at least 150 minutes of moderate intensity or at least 75 minutes of vigorous physical activity or equivalent combination of both. | Yes, meet recommendations No | 2 | Type 2 | Calculated from long IPAQ and GPAQ. |  |
| Physical Activity | SVYPAVLEI | Vigorous physical activities carried out solely for leisure time (recreation, sport, exercise or leisure activities): Minutes per week | Numeric | 2 | Type 2 | This is the vigorous leisure physical activity component from the leisure physical activity measure. Calculated from long IPAQ and GPAQ. |  |
| Physical Activity | SVYPAMLEI | Moderate physical activities carried out solely for leisure time (recreation, sport, exercise or leisure activities): Minutes per week | Numeric | 2 | Type 2 | This is the moderate leisure physical activity component from the leisure physical activity measure. Calculated from long IPAQ and GPAQ. |  |
| Physical Activity | SVYPATRAN | Transportation physical activity as defined as physical activity from cycling and walking undertaken to go from place to place (transportation): Minutes per week | Numeric | 2 | Type 2 | Calculated from long IPAQ and GPAQ. |  |
| Physical Activity | SVYPATRANCAT | Meeting recommendations of meeting physical activity recommendations via transportation physical activity. Meeting recommendations is defined as at least 150 minutes of moderate intensity or at least 75 minutes of vigorous physical activity or equivalent combination of both. | Yes, meet recommendations No | 2 | Type 2 | Calculated from long IPAQ and GPAQ. |  |
| Physical Activity | SVYPAWALK | Walking for leisure and/or transportation: Minutes per week | Numeric | 2 | Type 2 | Calculated from short IPAQ and long IPAQ. |  |
| Physical Activity | SVYPAWALKCAT | Meeting recommendations of meeting physical activity recommendations via walking for leisure and/or transportation. Meeting recommendations is defined as at least 150 minutes of moderate intensity or at least 75 minutes of vigorous physical activity or equivalent combination of both. | Yes, meet recommendations No | 2 | Type 2 | Calculated from short IPAQ and long IPAQ. |  |
| Physical Activity | SVYPAWLEI | Walking for leisure: Minutes per week | Numeric | 1 | Type 1 | Calculated from long IPAQ. |  |
| Physical Activity | SVYPAWTRN | Walking for transportation: Minutes per week | Numeric | 1 | Type 1 | Calculated from long IPAQ. |  |
| Physical Activity | SVYPAGLOB | Global physical activity for leisure and/or transportationn including moderate, vigorous, and walking: Minutes per week | Numeric | 3 | Type 2 | Calculated from short IPAQ, long IPAQ, and GPAQ. |  |
| Physical Activity | SVYPAGLOBCAT | Meeting recommendations of meeting physical activity recommendations via global physical activity. Meeting recommendations is defined as at least 150 minutes of moderate intensity or at least 75 minutes of vigorous physical activity or equivalent combination of both. | Yes, meet recommendations No | 3 | Type 2 | Calculated from short IPAQ, long IPAQ, and GPAQ. |  |
| Physical Activity | SVYPAVIG | Vigorous physical activity for leisure and/or transportation: Minutes per week | Numeric | 3 | Type 2 | This is the vigorous physical activity component from the global physical activity measure. Calculated from short IPAQ, long IPAQ, and GPAQ. |  |
| Physical Activity | SVYPAMOD | Moderate physical activity for leisure and/or transportation: Minutes per week | Numeric | 3 | Type 2 | This is the moderate physical activity component from the global physical activity measure. Calculated from short IPAQ, long IPAQ, and GPAQ. |  |
| Diet | SVYFRUDYWK1 | Number of days per week that fruit is consumed. | Numeric | 6 | Type 2 | Food and beverage consumption were assessed either through a food frequency questionnaire (FFQ) or non-FFQ screening questions. An FFQ is composed of a food and beverage list with response categories indicating the frequency of consuming each individual food or beverage over a specified time period. Because each food and beverage require a response independently, contextual information about intake (whether foods are consumed the same meal/day) are missing. In contrast, non-FFQ measures like screeners obtain some basic information about limited number of foods and beverages often grouped into food groups (fruits, vegetables etc.) |  |
| Diet | SVYVEGDYWK1 | Number of days per week that vegetables are consumed. | Numeric | 7 | Type 2 |  |  |
| Diet | SVYFVGDYWK1 | Number of days per week that fruits and/or vegetables are consumed. Sums number of days per week for fruit and vegetables variables and capped at 7 days if the sum is greater than 7. | Numeric | 7 | Type 2 |  |  |
| Diet | SVYFRUSVDY1 | Average number of servings of fruit per day on days an individual consumes fruit. | Numeric | 6 | Type 2 |  |  |
| Diet | SVYVEGSVDY1 | Average number of servings of vegetables per day on days an individual consumes vegetables. | Numeric | 6 | Type 2 |  |  |
| Diet | SVYFVGSVDY1 | Average number of servings of fruits and/or vegetables per day on days an individual consumes fruits and/or vegetables. Sums number of servings for fruit and vegetables variables. | Numeric | 6 | Type 2 |  |  |
| Diet | SVYSSBDYWK1 | Number of days per week sugar-sweetened beverages are consumed. | Numeric | 7 | Type 2 |  |  |
| Diet | SVYSSBSVDY1 | Average number of servings of sugar-sweetened beverages per day on days an individual consumes sugar-sweetened beverages. | Numeric | 7 | Type 2 |  |  |
| Diet | SVYSWTDYWK1 | Number of days per week sweet foods are consumed. | Numeric | 6 | Type 2 |  |  |
| Health Care | SVYPAPEVER | Ever received a pap test as defined as reporting having received a pap test at any time. Only reported amongst women. | Yes No | 2 | Type 2 |  | PAHO Cancer Country profile – 2013 (https://www.paho.org/en/file/36376/download?token=aqio9_6B), Argentina Ministry of Health (https://www.argentina.gob.ar/salud/cancer/prevencion), Peru Ministry of Health (https://cdn.www.gob.pe/uploads/document/file/337895/resolucion-ministerial-576-2019-minsa.PDF) |
| Health Care | SVYPAP3YRS | Received a pap test within last 3 years as defined as reporting having received a pap test within the last 3 years. Only reported amongst women. | Yes No | 6 | Type 2 | Three years was chosen due to most countries, except Peru, having country guidelines that women should be tested every 3 years. |  |
| Health Care | SVYMAMEVER | Ever received a mammogram as defined as reporting having received a mammogram at any time. Only reported amongst women. | Yes No | 2 | Type 2 |  |  |
| Health Care | SVYMAM2YRS | Received a mammogram within last 2 years as defined as reporting having received a mammogram within the last 2 years. Only reported amongst women. | Yes No | 5 | Type 2 | Two years was chosen due to most countries, except Peru, having no frequency guidelines. |  |
| Violence | SVYVIOLENCE | Experienced any type of violence or aggression in past 12 months. | Yes No | Each survey where available was different | Type 2 | The types of crime included as "violence or aggression" varies by survey. | World Health Organization (WHO) (https://www.who.int/violence_injury_prevention/violence/world_report/en/summary_en.pdf) |
| Violence | SVYVIOLN | Number of times the respondent experienced a violent event in past 12 months. | Numeric | Each survey where available was different | Type 2 |  |  |
| Violence | SVYFIREARM | Experienced violence involving firearms in past 12 months . | Yes No | Each survey where available was different | Type 2 |  |  |
| BMI: Body Mass Index  CDC: Center for Disease Control  CES-D SF: Center for Epidemiological Studies - Depression scale Short form  FFQ: Food Frequency Questionnaire  GPAQ: Global Physical Activity Questionnaire  IPAQ: International Physical Activity Questionnaire.  IPUMS: Integrated Public Use Microdata Series  kg/m2: Kilograms per meter squared  NHANES: U.S. Centers for Disease Control National Health and Nutrition Examination Survey  PAHO: Pan-American Health Organization  PHQ9: Patient Health Questionnaire  WHO: World Health Organization  %Question Scenarios. The questions asked on the survey were grouped into scenarios where surveys that the question was asked in the same way is considered a scenario. This indicates the total number of scenarios. In some cases, all surveys asked the questions to create the harmonized measures were different.  $Harmonization type. Harmonization methods fall into one of three types:  Type 1 Surveys had questions that were asked in the same way with only slight differences in wording such that there was only one question scenario. It was possible to create one harmonized version of the measure.  Type 2 The survey question was asked in different ways in term of wording, response options, and skip patterns available such that there were multiple question scenarios, but we were able to code in such a way to obtain one harmonized version of the measure.  Type 3 Multiple versions of the measure were created because we could not harmonize to a common definition due to differences in the question wording. | | | | | | | |

# Supplementary Table S3: Availability of harmonized variables included in Salud Urbana en America Latina/Urban Health in Latin America (SALURBAL) data

| **Variable Name** | **Number of adult surveys** | **Number of child surveys** | **Argentina** | **Brazil** | **Chile** | **Colombia** | **Costa Rica** | **Guatemala** | **Mexico** | **Nicaragua** | **Panama** | **Peru** | **El Salvador** |
| --- | --- | --- | --- | --- | --- | --- | --- | --- | --- | --- | --- | --- | --- |
| SVYMALE | 27 | 13 | 2005, 2009, 2013 | 2013, 2019 | 2003, 2010, 2017* | 2005*, 2007, 2010*, 2015* | 2005 | 2002, 2015* | 2000*, 2006*, 2012*, 2016*, 2018*, 2021* | 2003 | 2007 | 2016* | 2004, 2008*, 2014 |
| SVYAGE | 27 | 13 | 2005, 2009, 2013 | 2013, 2019 | 2003, 2010, 2017* | 2005*, 2007, 2010*, 2015* | 2005 | 2002, 2015* | 2000*, 2006*, 2012*, 2016*, 2018*, 2021* | 2003 | 2007 | 2016* | 2004, 2008*, 2014 |
| SVYAGEMO | NA | 13 |  |  | 2017* | 2005*, 2010*, 2015* |  | 2015* | 2000*, 2006*, 2012*, 2016*, 2018*, 2021* |  |  | 2016* | 2008* |
| SVYMARSTAT | 26 | 11 | 2005, 2009, 2013 | 2013, 2019 | 2003, 2010, 2017 | 2005*, 2007, 2010*, 2015* | 2005 | 2002, 2015 | 2000*, 2006*, 2012*, 2016*, 2018*, 2021* | 2003 | 2007 | 2016* | 2004, 2008* |
| SVYAFRODEC | 6 | 2 |  | 2013, 2019 |  | 2010*, 2015* |  | 2002 |  |  |  | 2016 |  |
| SVYINDIGENOUS | 12 | 5 |  | 2013, 2019 | 2017 | 2010*, 2015* |  | 2002, 2015 | 2006*, 2012*, 2016* | 2003 |  | 2016 |  |
| SVYDBDX1 | 20 | NA | 2005, 2009, 2013 | 2013, 2019 | 2003, 2010, 2017 | 2007 | 2005 | 2002 | 2006, 2012, 2016, 2018, 2021 | 2003 |  | 2016 | 2004, 2014 |
| SVYDBDX2 | 20 | NA | 2005, 2009, 2013 | 2013, 2019 | 2003, 2010, 2017 | 2007 |  | 2002 | 2000, 2006, 2012, 2016, 2018, 2021 | 2003 | 2007 |  | 2004, 2014 |
| SVYDBMED | 20 | NA | 2005, 2009, 2013 | 2013, 2019 | 2010, 2017 | 2007 | 2005 | 2002 | 2000, 2006, 2012, 2016, 2018, 2021 | 2003 |  | 2016 | 2004, 2014 |
| SVYHTDX1 | 13 | NA |  | 2013, 2019 | 2010, 2017 | 2007 | 2005 |  | 2006, 2012, 2016, 2018, 2021 |  |  | 2016 | 2014 |
| SVYHTDX2 | 21 | NA | 2005, 2009, 2013 | 2013, 2019 | 2003, 2010, 2017 | 2007 | 2005 | 2002 | 2000, 2006, 2012, 2016, 2018, 2021 | 2003 | 2007 |  | 2004, 2014 |
| SVYHTMED | 21 | NA | 2005, 2009, 2013 | 2013, 2019 | 2003, 2010, 2017 | 2007 | 2005 | 2002 | 2000, 2006, 2012, 2016, 2018, 2021 | 2003 |  | 2016 | 2004, 2014 |
| SVYHTMBP | 17 | NA |  | 2013 | 2003, 2010, 2017 | 2007 | 2005 | 2002 | 2000, 2006, 2012, 2016, 2018, 2021 | 2003 |  | 2016 | 2004, 2014 |
| SVYHTCTRL | 17 | NA |  | 2013 | 2003, 2010, 2017 | 2007 | 2005 | 2002 | 2000, 2006, 2012, 2016, 2018, 2021 | 2003 |  | 2016 | 2004, 2014 |
| SVYSBP | 17 | NA |  | 2013 | 2003, 2010, 2017 | 2007 | 2005 | 2002 | 2000, 2006, 2012, 2016, 2018, 2021 | 2003 |  | 2016 | 2004, 2014 |
| SVYDBP | 16 | NA |  |  | 2003, 2010, 2017 | 2007 | 2005 | 2002 | 2000, 2006, 2012, 2016, 2018, 2021 | 2003 |  | 2016 | 2004, 2014 |
| SVYWTKG | 23 | 13 |  | 2013, 2019 | 2003, 2010, 2017* | 2005*, 2007, 2010*, 2015* | 2005 | 2002, 2015* | 2000*, 2006*, 2012*, 2016*, 2018*, 2021* | 2003 |  | 2016* | 2004, 2008*, 2014 |
| SVYHTCM | 23 | 13 |  | 2013, 2019 | 2003, 2010, 2017* | 2005*, 2007, 2010*, 2015* | 2005 | 2002, 2015* | 2000*, 2006*, 2012*, 2016*, 2018*, 2021* | 2003 |  | 2016* | 2004, 2008*, 2014 |
| SVYBMI | 23 | 13 |  | 2013, 2019 | 2003, 2010, 2017* | 2005*, 2007, 2010*, 2015* | 2005 | 2002, 2015* | 2000*, 2006*, 2012*, 2016*, 2018*, 2021* | 2003 |  | 2016* | 2004, 2008*, 2014 |
| SVYBMIZ | N/A | 13 |  |  | 2017* | 2005*, 2010*, 2015* |  | 2015* | 2000*, 2006*, 2012*, 2016*, 2018*, 2021* |  |  | 2016* | 2008* |
| SVYHTAGZ | N/A | 13 |  |  | 2017* | 2005*, 2010*, 2015* |  | 2015* | 2000*, 2006*, 2012*, 2016*, 2018*, 2021* |  |  | 2016* | 2008* |
| SVYWTHTZ | N/A | 13 |  |  | 2017* | 2005*, 2010*, 2015* |  | 2015* | 2000*, 2006*, 2012*, 2016*, 2018*, 2021* |  |  | 2016* | 2008* |
| SVYWTAGZ | N/A | 13 |  |  | 2017* | 2005*, 2010*, 2015* |  | 2015* | 2000*, 2006*, 2012*, 2016*, 2018*, 2021* |  |  | 2016* | 2008* |
| SVYGRTHST | N/A | 13 |  |  | 2017* | 2005*, 2010*, 2015* |  | 2015* | 2000*, 2006*, 2012*, 2016*, 2018*, 2021* |  |  | 2016* | 2008* |
| SVYWTKGSELF | 6 | NA | 2005, 2009, 2013 | 2013, 2019 |  | 2007 |  |  |  |  |  |  |  |
| SVYHTCMSELF | 6 | NA | 2005, 2009, 2013 | 2013, 2019 |  | 2007 |  |  |  |  |  |  |  |
| SVYBMISELF | 6 | NA | 2005, 2009, 2013 | 2013, 2019 |  | 2007 |  |  |  |  |  |  |  |
| SVYHLTHSTAT1 | 9 | NA | 2005, 2009, 2013 |  | 2010 | 2005, 2010 |  | 2002 |  | 2003 |  |  | 2004 |
| SVYHLTHSTAT2 | 6 | NA |  | 2013, 2019 | 2017 | 2007 |  |  | 2000, 2006 |  |  |  |  |
| SVYHLTHSTAT3 | 1 | NA |  |  |  |  |  |  | 2012 |  |  |  |  |
| SVYCIGHX1 | 12 | NA | 2005, 2009, 2013 |  | 2003, 2010, 2017 | 2007 |  |  | 2000, 2006, 2012, 2016, 2018 |  |  |  |  |
| SVYCIGHX2 | 4 | NA | 2005,2009, 2013 |  |  |  |  |  |  |  |  |  | 2014 |
| SVYTOBHX1 | 4 | NA |  |  |  | 2007 | 2005 | 2002 |  |  |  |  | 2004 |
| SVYTOBHX2 | 5 | NA |  | 2013, 2019 |  |  |  |  | 2021 |  | 2007 |  | 2014 |
| SVYCIGCUR1 | 12 | NA | 2005, 2009, 2013 |  | 2003, 2010, 2017 | 2007 |  |  | 2000, 2006, 2012, 2016, 2018 |  |  |  |  |
| SVYCIGCUR2 | 10 | NA | 2005, 2009, 2013 | 2013, 2019 |  | 2010 |  | 2015 | 2021 |  |  | 2016 | 2008 |
| SVYTOBCUR1 | 5 | NA |  |  |  | 2007 | 2005 | 2002 |  | 2003 |  |  | 2004 |
| SVYTOBCUR2 | 5 | NA |  | 2013, 2019 |  |  |  |  | 2021 |  | 2007 |  | 2014 |
| SVYCURRALC1 | 18 | NA | 2005, 2009, 2013 | 2013, 2019 | 2010, 2017 | 2007 | 2005 | 2002, 2015 | 2000, 2006, 2012 | 2003 |  | 2016 | 2004, 2014 |
| SVYCURRALC2 | 10 | NA | 2009, 2013 |  | 2010, 2017 |  |  | 2002 | 2012 | 2003 | 2007 | 2016 | 2014 |
| SVYBINGE1 | 8 | NA | 2005, 2009, 2013 | 2013 |  |  |  |  | 2012, 2021 |  |  | 2016 | 2014 |
| SVYBINGE2 | 9 | NA | 2005, 2009, 2013 | 2019 |  |  |  | 2002 | 2006, 2016, 2018 |  |  | 2016 |  |
| SVYBINGE3 | 1 | NA |  |  |  | 2007 |  |  |  |  |  |  |  |
| SVYBINGE4 | 6 | NA | 2005, 2009, 2013 |  | 2010, 2017 |  |  |  |  |  |  | 2016 |  |
| SVYEVERPREG | 12 | NA |  | 2013 | 2010, 2017 | 2005 |  | 2015 | 2000, 2006, 2012, 2018, 2021 |  |  | 2016 | 2008 |
| SVYCURRPREG | 14 | NA |  | 2013, 2019 |  | 2005, 2010, 2015 |  | 2015 | 2000, 2012, 2018, 2021 |  | 2007 | 2016 | 2008, 2014 |
| SVYEDU | 27 | 12 | 2005, 2009, 2013 | 2013, 2019 | 2003, 2010, 2017* | 2005*, 2007, 2010*, 2015* | 2005 | 2002, 2015* | 2000*, 2006*, 2012*, 2016*, 2018*, 2021* | 2003 | 2007 | 2016* | 2004, 2008*, 2014 |
| SVYHHEDU | 17 | 12 | 2005, 2009, 2013 | 2013, 2019 | 2017* | 2005*, 2010*, 2015* |  | 2015* | 2000*, 2006*, 2012*, 2016*, 2018*, 2021* |  |  | 2016* |  |
| SVYMTHEDU | N/A | 7 |  |  | 2017* | 2005*, 2010*, 2015* |  | 2015* |  |  |  | 2016* | 2008* |
| SVYFLOOR | 21 | 12 | 2005, 2009, 2013 | 2013, 2019 | 2010, 2017 | 2005*, 2007, 2010*, 2015* |  | 2002, 2015* | 2000*, 2006*, 2012*, 2016*, 2018*, 2021* |  |  | 2016* | 2008* |
| SVYWALLDUR1 | 17 | 11 |  | 2013, 2019 | 2010, 2017 | 2005*, 2007, 2010*, 2015* |  | 2002, 2015* | 2000*, 2012*, 2016*, 2018*, 2021* |  |  | 2016* | 2008* |
| SVYWALLDUR2 | 16 | 10 |  | 2013, 2019 | 2010, 2017 | 2007, 2010*, 2015* |  | 2002, 2015* | 2000*, 2012*, 2016*, 2018*, 2021* |  |  | 2016* | 2008* |
| SVYWALLDUR3 | 18 | 12 |  | 2013, 2019 | 2010, 2017 | 2005*, 2007, 2010*, 2015* |  | 2002, 2015* | 2000*, 2006*, 2012*, 2016*, 2018*, 2021* |  |  | 2016* | 2008* |
| SVYNPER3RM | 14 | 7 | 2005, 2009, 2013 | 2013 |  | 2007 |  |  | 2000*, 2006*, 2012*, 2016*, 2018*, 2021* | 2003 |  | 2016* | 2004 |
| SVYNPER25BR | 19 | 13 | 2005, 2009, 2013 | 2013, 2019 | 2017* | 2005*, 2007, 2010*, 2015* |  | 2015* | 2000*, 2006*, 2012*, 2016*, 2018*, 2021* |  |  | 2016* | 2008* |
| SVYSEWANY | 21 | 13 | 2005, 2009, 2013 | 2013, 2019 | 2010, 2017* | 2005*, 2007, 2010*, 2015* |  | 2015* | 2000*, 2006*, 2012*, 2016*, 2018*, 2021* |  | 2007 | 2016* | 2008* |
| SVYSEWNET | 21 | 13 | 2005, 2009, 2013 | 2013, 2019 | 2010, 2017* | 2005*, 2007, 2010*, 2015* |  | 2015* | 2000*, 2006*, 2012*, 2016*, 2018*, 2021* |  | 2007 | 2016* | 2008* |
| SVYWATPIPE | 15 | 9 | 2005, 2009, 2013 | 2019 | 2010, 2017 |  |  | 2015* | 2000*, 2006*, 2012*, 2016*, 2018*, 2021* |  |  | 2016* | 2008* |
| SVYWATIN | 14 | 8 | 2005, 2009, 2013 | 2019 | 2010, 2017 |  |  | 2015* | 2000*, 2012*, 2016*, 2018*, 2021* |  |  | 2016* | 2008* |
| SVYWATNET | 20 | 13 | 2005, 2009, 2013 | 2013, 2019 | 2010, 2017* | 2005*, 2007, 2010*, 2015* |  | 2015* | 2000*, 2006*, 2012*, 2016*, 2018*, 2021* |  |  | 2016* | 2008* |
| SVYHHMALE | 19 | 13 | 2005, 2009, 2013 | 2013, 2019 | 2017* | 2005*, 2007, 2010*, 2015* |  | 2015* | 2000*, 2006*, 2012*, 2016*, 2018*, 2021* |  |  | 2016* | 2008* |
| SVYOWNCAR | 17 | 12 |  | 2013, 2019 | 2003, 2010 | 2005*, 2007, 2010*, 2015* |  | 2015* | 2000*, 2006*, 2012*, 2016*, 2018*, 2021* |  |  | 2016* | 2008* |
| SVYPHQ9A | 3 | NA |  | 2013, 2019 |  |  |  |  |  |  |  | 2016 |  |
| SVYPHQ9B | 3 | NA |  | 2013, 2019 |  |  |  |  |  |  |  | 2016 |  |
| SVYPHQ9C | 3 | NA |  | 2013, 2019 |  |  |  |  |  |  |  | 2016 |  |
| SVYPHQ9D | 3 | NA |  | 2013, 2019 |  |  |  |  |  |  |  | 2016 |  |
| SVYPHQ9E | 3 | NA |  | 2013, 2019 |  |  |  |  |  |  |  | 2016 |  |
| SVYPHQ9F | 3 | NA |  | 2013, 2019 |  |  |  |  |  |  |  | 2016 |  |
| SVYPHQ9G | 3 | NA |  | 2013, 2019 |  |  |  |  |  |  |  | 2016 |  |
| SVYPHQ9H | 3 | NA |  | 2013, 2019 |  |  |  |  |  |  |  | 2016 |  |
| SVYPHQ9I | 3 | NA |  | 2013, 2019 |  |  |  |  |  |  |  | 2016 |  |
| SVYPHQ9 | 3 | NA |  | 2013, 2019 |  |  |  |  |  |  |  | 2016 |  |
| SVYCESDC | 3 | NA |  |  |  |  |  |  | 2012, 2018, 2021 |  |  |  |  |
| SVYCESDE | 3 | NA |  |  |  |  |  |  | 2012, 2018, 2021 |  |  |  |  |
| SVYCESDF | 3 | NA |  |  |  |  |  |  | 2012, 2018, 2021 |  |  |  |  |
| SVYCESDG | 3 | NA |  |  |  |  |  |  | 2012, 2018, 2021 |  |  |  |  |
| SVYCESDK | 3 | NA |  |  |  |  |  |  | 2012, 2018, 2021 |  |  |  |  |
| SVYCESDP | 3 | NA |  |  |  |  |  |  | 2012, 2018, 2021 |  |  |  |  |
| SVYCESDR | 3 | NA |  |  |  |  |  |  | 2012, 2018, 2021 |  |  |  |  |
| SVYCESD7 | 3 | NA |  |  |  |  |  |  | 2012, 2018, 2021 |  |  |  |  |
| SVYDXDEP | 8 | NA |  | 2013, 2019 | 2010, 2017 |  |  |  | 2006, 2012, 2021 |  | 2007 |  |  |
| SVYDEPTX | 6 | NA |  | 2013, 2019 |  |  |  |  | 2006, 2012, 2021 |  |  | 2016 |  |
| SVYDEPMED | 4 | NA |  | 2013, 2019 | 2010, 2017 |  |  |  |  |  |  |  |  |
| SVYSRDEP1 | 3 | NA | 2005, 2009, 2013 |  |  |  |  |  |  |  |  |  |  |
| SVYSRDEP2 | 10 | NA |  | 2013, 2019 | 2003, 2010, 2017 |  |  |  | 2012, 2018, 2021 |  | 2007 | 2016 |  |
| SVYPALEIS | 9 | NA |  | 2013, 2019 | 2010, 2017 | 2005, 2010, 2015 |  | 2002 |  |  |  |  | 2014 |
| SVYPALEISCAT | 9 | NA |  | 2013, 2019 | 2010, 2017 | 2005, 2010, 2015 |  | 2002 |  |  |  |  | 2014 |
| SVYPAVLEI | 6 | NA |  |  | 2010, 2017 | 2005, 2010, 2015 |  | 2002 |  |  |  |  |  |
| SVYPAMLEI | 6 | NA |  |  | 2010, 2017 | 2005, 2010, 2015 |  | 2002 |  |  |  |  |  |
| SVYPATRAN | 9 | NA |  | 2013, 2019 | 2010, 2017 | 2005, 2010, 2015 |  | 2002 |  |  |  |  | 2014 |
| SVYPATRANCAT | 9 | NA |  | 2013, 2019 | 2010, 2017 | 2005, 2010, 2015 |  | 2002 |  |  |  |  | 2014 |
| SVYPAWALK | 12 | NA | 2005, 2009, 2013 |  |  | 2005, 2010, 2015 |  | 2002 | 2006, 2012, 2016, 2018 |  |  |  | 2014 |
| SVYPAWALKCAT | 12 | NA | 2005, 2009, 2013 |  |  | 2005, 2010, 2015 |  | 2002 | 2006, 2012, 2016, 2018 |  |  |  | 2014 |
| SVYPAWLEI | 4 | NA |  |  |  | 2005, 2010, 2015 |  | 2002 |  |  |  |  |  |
| SVYPAWTRN | 4 | NA |  |  |  | 2005, 2010, 2015 |  | 2002 |  |  |  |  |  |
| SVYPAGLOB | 11 | NA | 2005, 2009, 2013 |  | 2010, 2017 |  |  | 2002 | 2006, 2012, 2016, 2018 |  |  |  | 2014 |
| SVYPAGLOBCAT | 11 | NA | 2005, 2009, 2013 |  | 2010, 2017 |  |  | 2002 | 2006, 2012, 2016, 2018 |  |  |  | 2014 |
| SVYPAVIG | 11 | NA | 2005, 2009, 2013 |  | 2010, 2017 |  |  | 2002 | 2006, 2012, 2016, 2018 |  |  |  | 2014 |
| SVYPAMOD | 11 | NA | 2005, 2009, 2013 |  | 2010, 2017 |  |  | 2002 | 2006, 2012, 2016, 2018 |  |  |  | 2014 |
| SVYFRUDYWK1 | 16 | NA | 2005, 2009, 2013 | 2013, 2019 | 2010, 2017 | 2010, 2015 |  | 2002 | 2006, 2012, 2018, 2021 |  |  | 2016 | 2014 |
| SVYVEGDYWK1 | 16 | NA | 2005, 2009, 2013 | 2013, 2019 | 2010, 2017 | 2010, 2015 |  | 2002 | 2006, 2012, 2018, 2021 |  |  | 2016 | 2014 |
| SVYFVGDYWK1 | 17 | NA | 2005, 2009, 2013 | 2013, 2019 | 2003, 2010, 2017 | 2010, 2015 |  | 2002 | 2006, 2012, 2018, 2021 |  |  | 2016 | 2014 |
| SVYFRUSVDY1 | 16 | NA | 2009, 2013 | 2013 | 2010, 2017 | 2010, 2015 |  | 2002 | 2006, 2012, 2018, 2021 | 2003 |  | 2016 | 2004, 2014 |
| SVYVEGSVDY1 | 16 | NA | 2009, 2013 | 2013 | 2010, 2017 | 2010, 2015 |  | 2002 | 2006, 2012, 2018, 2021 | 2003 |  | 2016 | 2004, 2014 |
| SVYFVGSVDY1 | 16 | NA | 2009, 2013 | 2013 | 2010, 2017 | 2010, 2015 |  | 2002 | 2006, 2012, 2018, 2021 | 2003 |  | 2016 | 2004, 2014 |
| SVYSSBDYWK1 | 9 | NA |  | 2013, 2019 |  | 2010, 2015 |  | 2002 | 2006, 2012, 2018, 2021 |  |  |  |  |
| SVYSSBSVDY1 | 10 | NA |  | 2013 | 2017 | 2010, 2015 |  | 2002 | 2006, 2012, 2018, 2021 |  |  |  | 2014 |
| SVYSWTDYWK1 | 9 | NA |  | 2013, 2019 |  | 2010, 2015 |  | 2002 | 2006, 2012, 2018, 2021 |  |  |  |  |
| SVYPAPEVER | 12 | NA | 2005, 2009, 2013 | 2013 | 2003, 2010, 2017 | 2005 |  | 2015 | 2012 |  |  | 2016 | 2008 |
| SVYPAP3YRS | 11 | NA | 2009, 2013 | 2013 | 2003, 2010, 2017 | 2005 |  | 2015 | 2012 |  |  | 2016 | 2008 |
| SVYMAMEVER | 10 | NA | 2005, 2009, 2013 | 2013 | 2010, 2017 | 2005 |  |  | 2012 |  |  | 2016 | 2008 |
| SVYMAM2YRS | 10 | NA | 2005, 2009, 2013 | 2013 | 2010, 2017 | 2005 |  |  | 2012 |  |  | 2016 | 2008 |
| SVYVIOLENCE | 6 | NA | 2005, 2009 | 2013 | 2010 | 2007 |  |  | 2021 |  |  |  |  |
| SVYVIOLN | 1 | NA |  |  |  | 2007 |  |  |  |  |  |  |  |
| SVYFIREARM | 5 | NA | 2005, 2009 | 2013 |  | 2007 |  |  | 2021 |  |  |  |  |

*Indicates the years a child survey is available

# Supplementary Figure S5: Estimated prevalence of overweight for Salud Urbana en America Latina/Urban Health in Latin America (SALURBAL) cities and countries, by sex. (standardized to the pooled 2010 SALURBAL population age distribution)

AR: Argentina, BR: Brazil, CL: Chile, CO: Colombia, CR: Costa Rica, GT: Guatemala, MX: Mexico, NI: Nicaragua, PA: Panama, PE: Peru, SV: El Salvador

# Supplementary Figure S6: Estimated prevalence of diabetes for Salud Urbana en America Latina/Urban Health in Latin America (SALURBAL) cities and countries, by sex. (standardized to the pooled 2010 SALURBAL population age distribution)

AR: Argentina, BR: Brazil, CL: Chile, CO: Colombia, CR: Costa Rica, GT: Guatemala, MX: Mexico, NI: Nicaragua, PA: Panama, PE: Peru, SV: El Salvador

# Supplementary Figure S7: Estimated prevalence of hypertension for Salud Urbana en America Latina/Urban Health in Latin America (SALURBAL) cities and countries, by sex. (standardized to the pooled 2010 SALURBAL population age distribution)

AR: Argentina, BR: Brazil, CL: Chile, CO: Colombia, CR: Costa Rica, GT: Guatemala, MX: Mexico, NI: Nicaragua, PA: Panama, PE: Peru, SV: El Salvador

# Supplementary Figure S8: Estimated prevalence of poor-fair self reported health for Salud Urbana en America Latina/Urban Health in Latin America (SALURBAL) cities and countries, by sex. (standardized to the pooled 2010 SALURBAL population age distribution)

AR: Argentina, BR: Brazil, CL: Chile, CO: Colombia, CR: Costa Rica, GT: Guatemala, MX: Mexico, NI: Nicaragua, PA: Panama, PE: Peru, SV: El Salvador

# Supplementary Figure S9: Estimated prevalence of smoking for Salud Urbana en America Latina/Urban Health in Latin America (SALURBAL) cities and countries, by sex. (standardized to the pooled 2010 SALURBAL population age distribution)

AR: Argentina, BR: Brazil, CL: Chile, CO: Colombia, CR: Costa Rica, GT: Guatemala, MX: Mexico, NI: Nicaragua, PA: Panama, PE: Peru, SV: El Salvador

# Supplementary Figure S10: Estimated prevalence of adult (20 years and older) obesity over time in 46 selected Salud Urbana en America Latina/Urban Health in Latin America (SALURBAL) Mexican cities, by sex (standardized to the Mexico population age distribution).

**Each thin line corresponds to one of the 46 cities, and red-dotted line represents estimated country-level prevalence.**

**
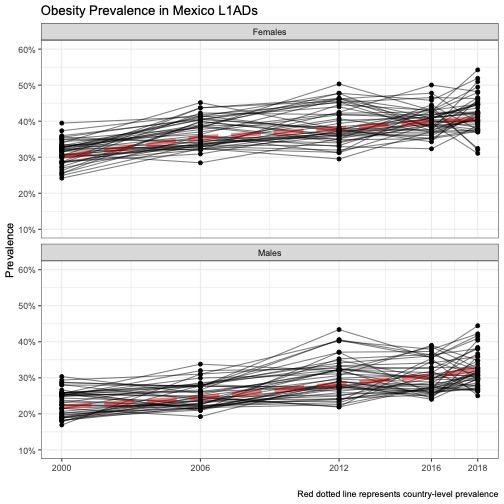
**

# Supplementary table S4. Selected characteristics of SALURBAL cities. All Salud Urbana en America Latina/Urban Health in Latin America (SALURBAL) cities and SALURBAL cities with at least one survey available and included in this resource.

|  | Sample with Adult Health Survey data | Sample with Children Health  Survey data | SALURBAL City Universe |
| --- | --- | --- | --- |
| Number of countries | 11  (AR, BR, CL, CO, CR, GT, MX, NI, PA, PE, SV) | 6  (CL, CO, GT, MX, PE, SV) | 11 |
| Number of cities | 240 | 134 | 371 |
| Median (p25-p75) total population in ‘000 (City size) | 352.3  (194.94-855.63) | 387.53  (225.19-814.91) | 280.50  (165.156, 547.95) |
| Median (p25-p75) Population density | 6894.95  (5293.12-9958.07) | 7743.22  (5727.42-12475.35) | 6342.28  (5040.06, 8694.27) |
| Mean (SD) % Households with overcrowding (Bedroom) | 19.15  (9.15) ^a^ | 22.25  (8.44) ^a^ | 15.90  (9.02) |
| Mean (SD) % Households with overcrowding | 7.45  (4.24) ^b^ | 8.32  (3.82) ^b^ | 5.69  (4.30) |
| Mean (SD) % population 25 and older with at least primary education | 79.31  (7.8) | 80.44  (7.22) | 73.80  (10.75) |
| Mean (SD) % households with sewage network | 71.71  (21.3) ^c^ | 78.04  (18.24) ^c^ | 68.76  (24.53) |

AR: Argentina, BR: Brazil, CL: Chile, CO: Colombia, CR: Costa Rica, GT: Guatemala, MX: Mexico, NI: Nicaragua, PA: Panama, PE: Peru, SV: El Salvador

p25: 25^th^ Percentile, p75: 7^th^ Percentile, SD: Standard Deviation

^a^ For adult survey sample, based on 216 cities (data unavailable for 23 PE and 1 NI cities); for child survey sample , based on 111 cities (data unavailable for 23 PE cities)

^b^ For adult survey sample, based on 218 cities (data unavailable for 21 CL and 1 NI cities); for child survey sample, based on 128 cities (data unavailable for 6 CL cities)

^c^ For adult survey sample, based on 219 cities (data unavailable for 21 CL); for child survey sample, based on 128 cities (data unavailable for 6 CL cities)

Data source for covariates: For AR, BR, MX, PA: all variables from 2010. For GT, population/population density from 2010 and others from 2018. For NI and CO, all variables from 2005. For CL and PE, all variables from 2017. For SV, all variables from 2007. For CR, all variables from 2011. Variables selected are from the most recent census data available at the time of writing.

# Methods for city-level prevalence estimates

While most of the surveys included in Salud Urbana en America Latina/Urban Health in Latin America (SALURBAL) were based on complex, multi-stage probability designs to select nationally representative samples of the population of the country and provide nationally representative estimates, these data cannot be used directly to draw inferences about prevalence of diseases or exposures in other geographic units, for example SALURBAL cities. For descriptive purposes and for potential utility by local public health actors SALURBAL generated estimates of the prevalence of diseases and risk factors in SALURBAL cities. We examined two potential statistical approaches: 1) Creation of post-stratification weights to re-weight the city-level survey samples to be representative and 2) Model-based, smoothed standardized estimates. Post-stratification weights were not possible to implement because the number of survey responses within some age-sex strata were very small in some cities and the full information about survey sampling was not available to our study team for all countries.

**Step 1: Modeling details**

We use a Bayesian hierarchical model to obtain smoothed estimates of prevalence for each survey outcome by sex and age, for each city. These models use a similar approach as Quick 2020. Models were run separately for each outcome, and separate models for each country. At this stage, only data from health surveys are used (population counts come later). Each model included all survey respondents with non-missing values for the outcome and age and sex categories. We used seven age categories as: 20-29, 30-39, 40-49, 50-59, 60-69, 70-79, and 80-89. Because not all surveys sampled persons under the age of 20, we excluded survey respondents under 20 years of age. We excluded respondents ages 90 and over due to small sample sizes in all surveys. We used two categories for sex (male and female).

Let $Y$ represent a binary outcome (e.g. obesity, smoking, etc.) and let $a$ denote age group {$a=1,\ldots,N_{a}; N_{a}=7\} total number of age groups considered as 10-year age groups$}, and $s$ represent sex{$s=1,\ldots,N_{s}$; $N_{s}=$ $2$ considering only male and female}. Let us assume that the outcome, $y$, for the $j^{th}$ individual, living in city $i$ {$i=1,\ldots, N_{i}$}, who is in age group $a$, and has sex $s$, has a Bernoulli distribution:

$$y_{j,ias} \sim Bernoulli\left( \theta_{ias} \right)$$

where $\theta_{ias}$ denotes the probability that outcome $y_{j,ias}=1$ and thus $\theta_{ias}$ represents the prevalence of the outcome for age group *a* and sex *s* in city *i*.

To model $\theta_{ias}$, we assume a logistic regression model of the form:

$$logit \theta_{ias} =logit\left( P\left( y_{j,ias}= 1 | \boldsymbol{\beta} , \boldsymbol{z} \right) \right)= \beta_{as} +z_{is},$$

where $\beta_{as}$ are intercept parameters for each combination of age and sex and $z_{is}$ is a random effect that varies by city and sex (sex- and city- specific values). Note that there is no grand-intercept; the model is coded in a ‘cell means’ fashion.

Although the surveys cover a large number of subjects, we expect that the sample size used for estimating each group-specific parameter will be small for some groups (e.g., elderly males if the survey had random selection, or young individuals if the survey oversampled older adults). These small sample sizes could lead to potentially unstable estimates of $\beta_{as}$ for those groups. We address this by defining appropriate prior formulations. For instance, we assign covariance structures to the intercept vector $\boldsymbol{\beta.}_{s}$ which allow sex groups to be correlated across cities to ‘borrow strength’ from each other (i.e., ‘smoothing’). We further assume that the differences in prevalence by sex is same for all age groups and then we let the intercepts deviate from there, essentially smoothing across age-groups across cities within a sex group.

We model the intercepts as arising from a multivariate normal distribution:

$${\boldsymbol{\beta}.}_{s} \sim MVN(\boldsymbol{\mu}_{.s},\boldsymbol{G}_{s})$$

where $\boldsymbol{\beta}_{.s}=\left( \beta_{a1},\ldots,\beta_{{aN}_{s}} \right)$ denotes the vector of $N_{s}$ sex-specific intercept parameters for each age group. The mean vector $\boldsymbol{\mu}_{.\boldsymbol{s}}=\left( \mu_{1},\ldots,\mu_{N_{s}} \right)$ corresponds to sex-specific intercept estimates for each age.

We further assume that the elements $\mu_{as}$ of the vector $\boldsymbol{\mu}$ satisfies $\mu_{as}$ =$c_{s}\mu_{s}+\mu_{a},$ where $c_{s}$ = (-0.5, 0.5) for females and males, respectively. In other words, we a priori assume that the differences in prevalence by sex is same for all age groups and then we let the intercepts deviate from there. We specified all $\mu_{a}$ to have an uninformative uniform prior over a reasonable range, $\mu_{a}\sim Uniform(-5, 5)$ and $\mu_{s}$ to have a zero-mean Normal prior, $\mu_{s}\sim Normal(0, 1)$.

The $N_{s}\times N_{s}$ (i.e., 2×2) covariance matrix $\boldsymbol{G}_{s}$, assumes that the intercept estimates for each sex may be correlated. This covariance structure is shared by all age groups. Off-diagonal elements of the matrix $\boldsymbol{G}_{s}, g_{s,s^{'}},$ correspond to the covariance between sex $s$ and $s^{'}$ (i.e. males and females).

The prior for $\boldsymbol{G}_{s}$ is an inverse Wishart distribution with the scale matrix $\boldsymbol{G}_{0}$ and the degrees of freedom $\nu=2. \boldsymbol{G}_{0}$ is a 2$\times$2 matrix with the elements $g_{0;s,s^{'}}$ whose elements are chosen to reduce prior informativeness:

$$g_{0;s,s^{'}}=\left\{ \begin{matrix} 0, & \mathrm{if} s\neq s^{'} \\ 0.5, & \mathrm{if} s=s^{'} \end{matrix} \right.$$

The random effect term $z_{is}$ captures the city-level effect for each sex. That is, while the intercepts $\beta_{as}$ estimate the country-level effect of age within sex categories on the probability of the outcome, $z_{is}$ helps to capture deviations in prevalence of the outcome of interest among the cities within country. For instance, females of all age groups in *i^th^* city have higher prevalence (compared to the country average) when *z_i,s=female_* is a positive. Or, in other words, the age-prevalence pattern for sex *s* in city *i* follows the country-level age-prevalence pattern for that sex determined by the $\boldsymbol{\beta}$, but it is shifted up or down for each sex in each city according to $z_{is}$.

We use a similar approach to estimate the random effects as we did for the intercepts. We define vector of $N_{s}=2$ random effects $\boldsymbol{z}_{i\bullet}$ for each L1AD, having elements $z_{is}$ representing the random effect for each combination of L1AD and sex (i.e., cell means coding).

The vector of random effects $\boldsymbol{z}_{i\bullet}$ with terms $z_{is}$ is assumed to have bivariate normal distribution, centered around a $N_{s}$ vector of zeros, with a covariance structure $\boldsymbol{\Gamma}$:

$$\boldsymbol{z}_{i}\sim MVN\left( \left( 0,\ldots,0 \right),\boldsymbol{\Gamma} \right)$$

The covariance structure $\boldsymbol{\Gamma}$ is a $N_{s}\times N_{s}$ matrix having elements $\gamma_{s,s^{'}}$ denoting the covariance between males and females (sex $s$ and $s^{'})$. The covariance structure $\boldsymbol{\Gamma}$ is assumed to have an inverse Wishart prior distribution on the matrix $\boldsymbol{\Gamma}_{0}$ and degrees of freedom $\nu=2$. The elements $\gamma_{0;s,s^{'}}$ of $\boldsymbol{\Gamma}_{0}$ are chosen to be minimally informative:

$$\gamma_{0;s,s^{'}} =\left\{ \begin{matrix} 0, & \mathrm{if} s\neq s^{'} \\ 1, & \mathrm{if} s=s^{'} \end{matrix} \right.$$

Each model was run in WinBUGS for 100,000 iterations for two chains. We discarded the initial 50,000 worth of samples for “burn-in” and thinned the remaining samples by a factor of 20. The results are based on final L = 5,000 posterior samples.

For the $\mathcal{l}^{th}$ iteration, where $\mathcal{l=}1,\ldots,L$, the model-based estimate of the prevalence for each city and age-sex group, based only on the survey data, $\hat{\theta}_{ias}^{\mathcal{(l)}}$, is computed from $\boldsymbol{\beta}^{\mathcal{(l)}}$ and $\boldsymbol{z}^{\mathcal{(l)}}$ using the formula:

$$\hat{\theta}_{ias}^{\mathcal{(l)}}=\frac{\exp\left( \beta_{as}^{\mathcal{(l)}} +z_{is}^{\mathcal{(l)}} \right)}{1+ \exp\left( \beta_{as}^{\mathcal{(l)}} +z_{is}^{\mathcal{(l)}} \right)}$$

***Modeling for Mammogram and Pap Test***

The mammogram and Pap test are female-specific outcomes and the model described above requires modification due to the measures only being available for females within a certain age group. Three age groups used in Pap test prevalence are 25-29, 30-39, and 40-49. The mammogram prevalence was calculated for age groups 50-59, 60-69, and 70-79.

Due to the data limitation in Peru ENDES 2016 survey, only two age groups are used for Pap Test (30-39, 40-49) and a single age group for mammogram (50-59). Since the Peru ENDES 2016 mammogram estimates involve a single age group, it is impossible to obtain the population-standardized prevalence rate and the estimate merely represents the prevalence among survey respondents.

Keeping the same denotation as above, we model the outcome $y$ for the $j^{th}$ individual of age group {$a=1,\ldots,N_{a}; N_{a}=3\}$ living in city $i$ {$i=1,\ldots, N_{i}$} as:

$$y_{j,ia} \sim Bernoulli\left( \theta_{ia} \right)$$

where $\theta_{ia}$ represents the prevalence of the outcome for age group *a* in city *i* and

$$logit \theta_{ia} =logit\left( P\left( y_{j,ia}= 1 | \boldsymbol{\beta} ,\boldsymbol{z} \right) \right)= \beta_{a} +z_{i}.$$

We specified all $\beta_{a}$ to have an uninformative uniform prior over a reasonable range, $\beta_{a}\sim Uniform(-15, 15)$ and $z_{i},$ to have a zero-mean Normal prior, $\mu_{i}\sim Normal(0,\sigma^{2})$

$$\sigma^{2}\sim Inv Gamma\left( 0.05,0.05 \right)$$

Post-model aggregation is performed in a similar manner (to be described in step 2) as other outcomes occurring in both sex with necessary adaptations. For example, prevalence of mammogram is standardized to female SALURBAL population 50-79 years.

***Modeling for Child outcomes***

The modeling approach for some child outcomes (1-4 years) is slightly different. We did not stratify the data by age and sex. Thus, to model the prevalence, we used the logistic regression of the form:

$$logit \theta_{i} =logit\left( P\left( y_{j,i}= 1 | \beta, z \right) \right)= \beta+z_{i},$$

where $z_{i}$ is random effect varying by city and is assumed to be distributed Normally with mean 0 and variance $\sigma^{2}$. We put a relatively uninformative Inverse-Gamma prior on variance and a flat (uniform over all real numbers) prior for $\beta$. That is,

$$\sigma^{2}\sim Inv Gamma\left( 0.05,0.005 \right)$$

$\beta\sim Uniform (-\infty$,$\infty)$

Since there is no age/sex group in the child outcomes, population standardization step is inapplicable. The estimates thus correspond to the estimates of prevalence among survey respondents.

**Step 2: Post-stratification/Post-model aggregation of prevalence/ Standardization of prevalence**

**Within-city standardization (Population representative crude rates)**

Obtaining prevalence rates that are representative at the city-level requires knowledge of the proportion of the population in the city within in each age-sex group. Let ${population}_{ias}$ be the population count in city $i=1,\ldots N_{i}$ for age group $a=1,\ldots N_{a}$, and sex $s=1,2$, as obtained from the population projection data from the same calendar year as the survey administration, and ${population}_{is} = \sum_{a=1}^{N_{a}} \left[ {population}_{ias} \right]$ be the population count for sex $s$ in city $i$. Here, $N_{i}$ is the number of cities in the country; and $N_{a}$ is the number of age categories in that country.

Crude sex-specific prevalence estimates for each city is:

$$\hat{\theta}_{is}^{CRUDE\mathcal{,(l)}}=\frac{\sum_{a=1}^{N_{a}} \left[ \hat{\theta}_{ias}^{\mathcal{(l)}}\times{population}_{ias} \right]}{\sum_{a=1}^{N_{a}} {population}_{ias}}$$

This CRUDE estimate is better than simply taking the observed proportion of cases in the survey data for a specific city, because it weighs the estimates to the population of the city. This is akin to post-stratification weights discussed in the survey literature.

Similarly, the *crude* city-specific estimate, combining sex groups, is:

$$\hat{\theta}_{i}^{CRUDE\mathcal{,(l)}}=\frac{\sum_{s=1}^{N_{s}} \sum_{a=1}^{N_{a}} \left[ \hat{\theta}_{ias}^{\mathcal{(l)}}\times{population}_{ias} \right]}{\sum_{s=1}^{N_{s}} \sum_{a=1}^{N_{a}} {population}_{ias}}$$

The estimates can be aggregated to the country-level for each sex, using:

$$\hat{\theta}_{\mathrm{Country},s}^{CRUDE\mathcal{,(l)}}=\frac{\sum_{i=1}^{N_{i}} \sum_{a=1}^{N_{a}} \left[ \hat{\theta}_{ias}^{\mathcal{(l)}}\times{population}_{ias} \right]}{\sum_{i=1}^{N_{i}} \sum_{a=1}^{N_{a}} {population}_{ias}}$$

or for the country combining sex groups:

$$\hat{\theta}_{\mathrm{Country}}^{CRUDE\mathcal{,(l)}}=\frac{\sum_{i=1}^{N_{i}} \sum_{a=1}^{N_{a}} \sum_{s=1}^{N_{s}} \left[ \hat{\theta}_{ias}^{\mathcal{(l)}}\times{population}_{ias} \right]}{\sum_{i=1}^{N_{i}} \sum_{a=1}^{N_{a}} \sum_{s=1}^{N_{s}} {population}_{ias}}$$

Because these calculations are performed for each of $\mathcal{l=}1, \ldots,L$ iterations, both a point estimate and an interval can be derived. Specifically, for each outcome prevalence estimate, the point estimate and lower and upper bounds of the 95% credible interval were determined by taking the 50^th^, 2.5^th^, and 97.5^th^ percentiles, respectively, of the estimates from all $L$ posterior samples.

**Within Country standardization**

We have operationalized within-country standardization in following two ways: (a) standardizing sex-specific prevalence in the city to sex-specific country population, and (b) standardizing sex-specific and sex-pooled prevalence in the city to sex-pooled country population.

***Standardization of sex-specific prevalence to country sex-specific population***

As above, let ${population}_{ias}$ be the population count in city $i=1,\ldots N_{i}$ for age group $a=1,\ldots N_{a}$, and sex $s=1,2$, as obtained from the population projection data from the same calendar year as the survey administration. The city-level prevalence estimates standardized to the population distribution of the country (i.e., adjusting for age and sex) are obtained from the model-based prevalence estimates $\hat{\theta}_{ias}^{\mathcal{(l)}}$ using population-based weighting, applying the following steps:

1. Calculate the country-level population proportions for each age group within sex.

$${Prop}_{\mathrm{Country},as}=\frac{{population}_{\mathrm{Country},as}}{{population}_{\mathrm{Country},s}}=\frac{\sum_{i=1}^{N_{i}} {population}_{ias}}{\sum_{a=1}^{N_{a}} \sum_{i=1}^{N_{i}} {population}_{ias}}$$

2. For each city, calculate adjusted population counts for each sex:

$${population}_{\mathrm{adjusted};ias}={population}_{is}\times{Prop}_{\mathrm{Country},as}=\frac{\left( \sum_{a=1}^{N_{a}} {population}_{ias} \right)\times\left( \sum_{i=1}^{N_{i}} {population}_{ias} \right)}{\left( \sum_{a=1}^{N_{a}} \sum_{i=1}^{N_{i}} {population}_{ias} \right)}$$

3. For each city, calculate sex-specific prevalence estimates, adjusted to the age-sex population structure of the country, $\hat{\theta}_{is}^{Country\mathcal{,(l)}}$:

$$\hat{\theta}_{is}^{Country\mathcal{,(l)}}=\frac{\sum_{a=1}^{N_{a}} \left[ \hat{\theta}_{ias}^{\mathcal{(l)}}\times{population}_{\mathrm{adjusted};ias} \right]}{\sum_{a=1}^{N_{a}} {population}_{\mathrm{adjusted};ias}}$$

***Standardization of prevalence to country sex-pooled population***

Keeping the same notation as above, let ${population}_{ias}$ be the population count in cities $i=1,\ldots N_{i}$ for age group $a=1,\ldots N_{a}$, and sex $s=1,2$, as obtained from the population projection data from the same calendar year as the survey administration.

1. Calculate the country-level population proportions for each age group for both sexes combined.

$${Prop}_{\mathrm{Country},a}=\frac{{population}_{\mathrm{Country},a}}{{population}_{\mathrm{Country}}}=\frac{\sum_{s=1}^{N_{s}} \sum_{i=1}^{N_{i}} {population}_{ias}}{\sum_{s=1}^{N_{s}} \sum_{a=1}^{N_{a}} \sum_{i=1}^{N_{i}} {population}_{ias}}$$

2. For each city, calculate adjusted population counts for each sex:

$${population}_{\mathrm{adjusted};ias}={population}_{is}\times{Prop}_{\mathrm{Country},a}=\frac{\left( \sum_{a=1}^{N_{a}} {population}_{ias} \right)\times(\sum_{s=1}^{N_{s}} \sum_{i=1}^{N_{i}} {population}_{ias})}{\sum_{s=1}^{N_{s}} \sum_{a=1}^{N_{a}} \sum_{i=1}^{N_{i}} {population}_{ias}}$$

3. For each city, calculate sex-specific prevalence estimates, adjusted to the age population structure of the country, $\hat{\theta}_{is}^{Country\mathcal{,(l)}}$:

$$\hat{\theta}_{is}^{Country\mathcal{,(l)}}=\frac{\sum_{a=1}^{N_{a}} \left[ \hat{\theta}_{ias}^{\mathcal{(l)}}\times{population}_{\mathrm{adjusted};ias} \right]}{\sum_{a=1}^{N_{a}} {population}_{\mathrm{adjusted};ias}}$$

4. For each city, compute the combined-sex (males and females) prevalence estimates standardized to the population structure of the country:

$$\hat{\theta}_{i}^{Country\mathcal{,(l)}}=\frac{\sum_{s=1}^{N_{s}} \sum_{a=1}^{N_{a}} \left[ \hat{\theta}_{ias}^{\mathcal{(l)}}\times{population}_{\mathrm{adjusted};ias} \right]}{\sum_{s=1}^{N_{s}} \sum_{a=1}^{N_{a}} {population}_{\mathrm{adjusted};ias}}$$

Furthermore, based on the prevalence of the cities, we can aggregate the prevalence up to country level.

Country-level sex-specific prevalence estimates:

$$\hat{\theta}_{\mathrm{Country},s}^{\mathcal{(l)}}=\frac{\sum_{i=1}^{N_{i}} \sum_{a=1}^{N_{a}} \left[ \hat{\theta}_{ias}^{\mathcal{(l)}}\times{population}_{ias} \right]}{\sum_{i=1}^{N_{i}} \sum_{a=1}^{N_{a}} {population}_{ias}}$$

Country-level combined-sex (males and females) prevalence estimates:

$$\hat{\theta}_{\mathrm{Country}}^{\mathcal{(l)}}=\frac{\sum_{i=1}^{N_{i}} \sum_{a=1}^{N_{a}} \sum_{s=1}^{N_{s}} \left[ \hat{\theta}_{ias}^{\mathcal{(l)}}\times{population}_{ias} \right]}{\sum_{i=1}^{N_{i}} \sum_{a=1}^{N_{a}} \sum_{s=1}^{N_{s}} {population}_{ias}}$$

**Between-country standardization**

Standardizing health outcome rates to the entire SALURBAL population enables comparisons of cities that are not in the same country. We have operationalized between-country standardization in following two ways: (a) standardizing sex-specific prevalence in the city to sex-specific SALURBAL 2010 population, and (b) standardizing sex-specific and sex-pooled prevalence in the city to sex-pooled SALURBAL 2010 population.

***Standardization of sex-specific prevalence to SALURBAL sex-specific population***

Let ${population}_{ias}$ be the population count in city $i=1,\ldots N_{i}$ for age group $a=1,\ldots N_{a}$, and sex $s=1,2$, as obtained from the population projections data for the year 2010, and ${population}_{is}$ be the population count for sex s in L1AD $i$. Here, $N_{i}$ is the number of L1ADs in the entire SALURBAL cities with data on the health outcome of interest; and $N_{a}$ is the number of age categories that are homogenous across countries.

City prevalence estimates are derived from the model-based prevalence estimates $\hat{\theta}_{ias}^{\mathcal{(l)}}$, obtained from country stratified models, and population-based weighting, applying the following steps. One could argue that a single model that includes data for all countries could be used, that includes random effects for countries. Utilizing the country-stratified model estimates, however, is likely more robust, because, for example, the stratified models also include interactions between country and age (not only the random effects for country).

1. Calculate the SALURBAL population proportions for each age group within sex.

$${Prop}_{\mathrm{SAL},as}=\frac{{population}_{\mathrm{SAL},as}}{{population}_{\mathrm{SAL},s}}=\frac{\sum_{i=1}^{N_{i}} {population}_{ias}}{\sum_{a=1}^{N_{a}} \sum_{i=1}^{N_{i}} {population}_{ias}}$$

2. For each city, calculate adjusted population counts for each sex:

$${population}_{\mathrm{SAL};ias}={population}_{is}\times{Prop}_{\mathrm{SAL},as}=\frac{\left( \sum_{a=1}^{N_{a}} {population}_{ias} \right)\times\left( \sum_{i=1}^{N_{i}} {population}_{ias} \right)}{\left( \sum_{a=1}^{N_{a}} \sum_{i=1}^{N_{i}} {population}_{ias} \right)}$$

3. For each city, calculate sex-specific prevalence estimates, adjusted to the age-sex population structure of SALURBAL cities, $\hat{\theta}_{is}^{\mathrm{SAL}\mathcal{,(l)}}$:

$$\hat{\theta}_{is}^{\mathrm{SAL}\mathcal{,(l)}}=\frac{\sum_{a=1}^{N_{a}} \left[ \hat{\theta}_{ias}^{\mathcal{(l)}}\times{population}_{\mathrm{SAL};ias} \right]}{\sum_{a=1}^{N_{a}} {population}_{\mathrm{SAL};ias}}$$

Furthermore, we can calculate the country-level, sex-specific prevalence standardized to SALURBAL 2010 sex-specific population as follows

Country-level sex-specific prevalence estimates:

$$\hat{\theta}_{\mathrm{Country},s}^{SAL\mathcal{(l)}}=\frac{\sum_{i=1}^{N_{i}(country)} \sum_{a=1}^{N_{a}} \left[ \hat{\theta}_{ias}^{\mathcal{(l)}}\times{population}_{\mathrm{SAL};ias} \right]}{\sum_{i=1}^{N_{i}(country)} \sum_{a=1}^{N_{a}} {population}_{\mathrm{SAL};ias}}$$

*NOTE: the outer sum is summing only over the cities in the specific country $N_{i}(country$)*

***Standardization of prevalence to SALURBAL sex-pooled population***

As above, Let ${population}_{ias}$ be the population count in city $i=1,\ldots N_{i}$ for age group $a=1,\ldots N_{a}$, and sex $s=1,2$, as obtained from the population projections data for the year 2010, and ${population}_{is}$ be the population count for sex s in city $i$. Here, $N_{i}$ is the number of cities in the entire SALURBAL cities with data on the health outcome of interest; and $N_{a}$ is the number of age categories that are homogenous across countries.

1. Calculate the SALURBAL population proportions for each age group (both sexes combined).

$${Prop}_{\mathrm{SAL},a}=\frac{{population}_{\mathrm{SAL},a}}{{population}_{\mathrm{SAL}}}=\frac{\sum_{s=1}^{N_{s}} \sum_{i=1}^{N_{i}} {population}_{ias}}{\sum_{s=1}^{N_{s}} \sum_{a=1}^{N_{a}} \sum_{i=1}^{N_{i}} {population}_{ias}}$$

2. For each city, calculate adjusted population counts for each sex:

$${population}_{\mathrm{SAL};ias}={population}_{is}\times{Prop}_{\mathrm{SAL},a}=\frac{\left( \sum_{a=1}^{N_{a}} {population}_{ias} \right)\times\sum_{s=1}^{N_{s}} \sum_{i=1}^{N_{i}} {population}_{ias}}{\sum_{s=1}^{N_{s}} \sum_{a=1}^{N_{a}} \sum_{i=1}^{N_{i}} {population}_{ias}}$$

1. For each city, calculate sex-specific prevalence estimates, adjusted to the age-sex population structure of SALURBAL cities, $\hat{\theta}_{is}^{\mathrm{SAL}\mathcal{,(l)}}$:

$$\hat{\theta}_{is}^{\mathrm{SAL}\mathcal{,(l)}}=\frac{\sum_{a=1}^{N_{a}} \left[ \hat{\theta}_{ias}^{\mathcal{(l)}}\times{population}_{\mathrm{SAL};ias} \right]}{\sum_{a=1}^{N_{a}} {population}_{\mathrm{SAL};ias}}$$

2. For each city, compute the combined-sex (males and females) prevalence estimates standardized to the population structure of the SALURBAL cities:

$$\hat{\theta}_{i}^{\mathrm{SAL}\mathcal{,(l)}}=\frac{\sum_{s=1}^{N_{s}} \sum_{a=1}^{N_{a}} \left[ \hat{\theta}_{ias}^{\mathcal{(l)}}\times{population}_{\mathrm{SAL};ias} \right]}{\sum_{s=1}^{N_{s}} \sum_{a=1}^{N_{a}} {population}_{\mathrm{SAL};ias}}$$

Furthermore, we can calculate the country-level prevalence standardized to SALURBAL 2010 population as follows:

Country-level sex-specific prevalence estimates:

$$\hat{\theta}_{\mathrm{Country},s}^{SAL\mathcal{(l)}}=\frac{\sum_{i=1}^{N_{i}(country)} \sum_{a=1}^{N_{a}} \left[ \hat{\theta}_{ias}^{\mathcal{(l)}}\times{population}_{\mathrm{SAL};ias} \right]}{\sum_{i=1}^{N_{i}(country)} \sum_{a=1}^{N_{a}} {population}_{\mathrm{SAL};ias}}$$

*NOTE: the outer sum is summing only over the cities in the specific country $N_{i}(country$)*

*C*ountry-level combined-sex (males and females) prevalence estimates:

$$\hat{\theta}_{\mathrm{Country}}^{SAL\mathcal{(l)}}=\frac{\sum_{i=1}^{N_{i}(country)} \sum_{a=1}^{N_{a}} \sum_{s=1}^{N_{s}} \left[ \hat{\theta}_{ias}^{\mathcal{(l)}}\times{population}_{\mathrm{SAL};ias} \right]}{\sum_{i=1}^{N_{i(country)}} \sum_{a=1}^{N_{a}} \sum_{s=1}^{N_{s}} {population}_{\mathrm{SAL};ias}}$$

Definitions of prevalence estimates available in SALURBAL

| **Description** | **Definition** | **Sex and Age Range** |
| --- | --- | --- |
| Diabetes Version 1 | A participant is described as having diabetes (diabetes mellitus) if they report having been told by a physician or health care provider that they have diabetes or high blood sugar levels. Women who report that a diabetes diagnosis was made during pregnancy are classified as NOT having diabetes. | Males and Females 20-89  (Age categories used: 20-29, 30-39,40-49,50-59,60-69,70-79, 80-89) |
| Diabetes Version 2 | A participant is described as having diabetes (diabetes mellitus or gestational diabetes) if they report having been told by a physician or health care provider that they have diabetes or high blood sugar levels, including women who report that this diagnosis was made during pregnancy | Males and Females 20-89 (Age categories used: 20-29, 30-39,40-49,50-59,60-69,70-79, 80-89) |
| Hypertension based on medication use | A participant is defined as hypertensive if they report that a physician told them that they had hypertension AND if they report using medications “to lower blood pressure” or control hypertension prescribed by a health care provider | Males and Females 20-89 (Age categories used: 20-29, 30-39,40-49,50-59,60-69,70-79, 80-89) |
| Adult Obesity [measured] | Obesity as defined as Body Mass Index >= 30 from measured height and weight | Males and Females 20-89 (Age categories used: 20-29, 30-39,40-49,50-59,60-69,70-79, 80-89)  Females 20-49 (Age categories used: 20-29, 30-39,40-49) |
| Adult Obesity [self-report] | Obesity as defined as Body Mass Index >= 30 from self-reported height and weight | Males and Females 20-89 (Age categories used: 20-29, 30-39,40-49,50-59,60-69,70-79, 80-89)  Females 20-49 (Age categories used: 20-29, 30-39,40-49) |
| Adult Overweight [measured] | Overweight as defined as Body Mass Index >= 25 from measured height and weight | Males and Females 20-89 (Age categories used: 20-29, 30-39,40-49,50-59,60-69,70-79, 80-89)  Females 20-49 (Age categories used: 20-29, 30-39,40-49) |
| Adult Overweight [self-report] | Overweight as defined as Body Mass Index >=25 from self-reported height and weight | Males and Females 20-89 (Age categories used: 20-29, 30-39,40-49,50-59,60-69,70-79, 80-89)  Females 20-49 (Age categories used: 20-29, 30-39,40-49) |
| Adult Underweight [measured] | Underweight as defined as Body Mass Index <18.5 from measured height and weight | Males and Females 20-89 (Age categories used: 20-29, 30-39,40-49,50-59,60-69,70-79, 80-89)  Females 20-49 (Age categories used: 20-29, 30-39,40-49) |
| Adult Underweight [self-report] | Underweight as defined as Body Mass Index <18.5 from self-reported height and weight | Males and Females 20-89 (Age categories used: 20-29, 30-39,40-49,50-59,60-69,70-79, 80-89)  Females 20-49 (Age categories used: 20-29, 30-39,40-49) |
| Child Stunting | Child stunting as defined as height-for-age z- score < -2 from measured height and age | Males and Females 1-4 |
| Child Wasting | Child wasting as defined as weight for height z-score < -2 from measured height and weight | Males and Females 1-4 |
| Child Overweight | Child overweight as defined as weight for height z-score >2 from measured height and weight | Males and Females 1-4 |
| Fair/Poor Self rated health | General self-reported health status dichotomized as reporting poor or fair health vs. good, very good, or excellent health | Males and Females 20-89 (Age categories used: 20-29, 30-39,40-49,50-59,60-69,70-79, 80-89) |
| Current Smoking | Current smoker of cigarettes or tobacco products | Males and Females 20-89 (Age categories used: 20-29, 30-39,40-49,50-59,60-69,70-79, 80-89) |
| Alcohol use within last 30 days | Current alcohol use version 1 is defined as drinking at least one alcoholic beverage in the past 30 days | Males and Females 20-89 (Age categories used: 20-29, 30-39,40-49,50-59,60-69,70-79, 80-89) |
| Alcohol use within last 12 months | Current alcohol use version 2 is defined as drinking at least one alcoholic beverage in the past 12 months | Males and Females 20-89 (Age categories used: 20-29, 30-39,40-49,50-59,60-69,70-79, 80-89) |
| Patient Health Questionnaire (PHQ-9) Depression indicator for major depression | PHQ-9 indicator for major depression, dichotomized as scores of 0-9 vs. 10-27 with sensitivity and specificity of 88% to detect major depression. This categorizes the PHQ-9 score into depression severity as determined by the PHQ-9 algorithm instructions | Males and Females 20-89 (Age categories used: 20-29, 30-39,40-49,50-59,60-69,70-79, 80-89) |
| Center for Epidemiology Studies – Depression Short Form (CESD-SF 7) questions depression category | CES-D SF 7 questions scale categorized as depressive symptoms (9-21) vs. no depressive symptoms (0-8) per validated cut points | Males and Females 20-89 (Age categories used: 20-29, 30-39,40-49,50-59,60-69,70-79, 80-89) |
| Doctor diagnosis of depression | A survey participant is classified as having depression if they report having been told by a physician or health care provider that they have depression | Males and Females 20-89 (Age categories used: 20-29, 30-39,40-49,50-59,60-69,70-79, 80-89) |
| Leisure physical activity: meeting recommendations | A cutoff point of 150 minutes per week was used to classify adults as active versus inactive, in accordance with the recommendations from World Health Organization (WHO), 2018 definition of active as at least 150 minutes of moderate-intensity physical activity throughout the week, or at least 75 minutes of vigorous-intensity physical activity throughout the week, or an equivalent combination of moderate- and vigorous-intensity activity | Males and Females 20-89 (Age categories used: 20-29, 30-39,40-49,50-59,60-69,70-79, 80-89) |
| Global physical activity: meeting recommendations | A cutoff point of 150 minutes per week was used to classify adults as active versus inactive, in accordance with the recommendations from WHO, 2018 definition of active as at least 150 minutes of moderate-intensity physical activity throughout the week, or at least 75 minutes of vigorous-intensity physical activity throughout the week, or an equivalent combination of moderate- and vigorous-intensity activity | Males and Females 20-89 (Age categories used: 20-29, 30-39,40-49,50-59,60-69,70-79, 80-89) |
| Pap test in last 3 years | Having received a pap test in the past three years | Females 25-49 (Age categories used: 25-29,30-39,40-49)  Females 30-49 (Age categories used: 30-39,40-49) |
| Mammogram in last 2 years | At least one mammography in the past two years | Females 50-79 (Age categories used:50-59,60-69,70-79) |

CESD-SF 7: Center for Epidemiology Studies – Depression Short Form

PHQ-9: Patient Health Questionnaire

# References

Trends in Tract-Level Prevalence of Obesity in Philadelphia by Race-Ethnicity, Space, and Time. Quick H, Terloyeva D, Wu X, Moore K, Diez Roux AV. Epidemiology. 2020 Jan; 31(1): 15-21.

Age Standardization of Death Rates: Implementation of the Year 2020 Standard. Anderson RN, Rosenberg HM. NVSR. 1998 Oct; 47(3): 1-20.
